# Glycosylated coumarins, flavonoids, lignans and phenylpropanoids from *Wikstroemia nutans* and their biological activities

**DOI:** 10.3762/bjoc.18.23

**Published:** 2022-02-16

**Authors:** Meifang Wu, Xiangdong Su, Yichuang Wu, Yuanjing Luo, Ying Guo, Yongbo Xue

**Affiliations:** 1 School of Pharmaceutical Sciences (Shenzhen), Sun Yat-sen University, No. 66 Gongchang Road, Shenzhen, 518107, China

**Keywords:** coumarin glucosides, flavonoids, lignans, structure elucidation, *Wikstroemia nutans*

## Abstract

*Wikstroemia nutans* Champ. ex Benth., a traditional herbal medicine collected at the Lingnan region of China, was chemically investigated. A new biscoumarin glucoside, wikstronutin (**1**), along with three known bis- and tricoumarin glucosides (**2**–**4**), two flavonoid glycosides (**5**–**6**), and eleven lignan glucosides (**7**–**17**) were isolated from the stems and roots of *W. nutans*. The new structure including its absolute configuration was elucidated based on a combination of 1D and 2D NMR, UV, IR, HRESIMS spectroscopic data, as well as chemical transformation. Compounds **1**–**17** were first isolated from the plant species *W. nutans*, while compounds **1**–**3**, **8**, and **11** were reported from the genus *Wikstroemia* for the first time. All co-isolates were evaluated for their in vitro inhibitory effects on nitric oxide (NO) production induced by lipopolysaccharide (LPS) in murine RAW264.7 macrophage cells. The antibacterial activity of the selected compounds was also tested. Our work enriches the structure diversity of the secondary metabolites from the genus *Wikstroemia.*

## Introduction

The genus *Wikstroemia* (Thymelaeaceae) contains approximately 62 species, which are widespread throughout the subtropical regions of Asia and Oceania [1]. Nineteen species of the genus *Wikstroemia* are found to be domestic in China, such as *W. nutans, W. indica*, *and W. canescens* [2]. Previous investigations have not only reported diverse secondary metabolites from the genus, but also promising pharmacological activities of the extracts and chemical constituents produced by *Wikstroemia* species, including cardiovascular, neuroprotective, hepatoprotective, anti-inflammatory, and antitumor activities [1,3]. The plant species *W. nutans* is widely distributed in the areas of the Guangdong and Guangxi provinces of China, and the whole plants of this species are used as a folk medicine for the treatment of arthritis, mastitis, and pain relief [2]. Interestingly, the traditional medical usages are highly consistent with the phytologically related medicinal plant species *W. indica* that has already been approved to use as a prescription drug in China [3]. Owing to the intriguing therapeutic effects associated with *W. indica*, extensive phytochemical studies on *W. indica* have been pursed [3–4]. However, there is no report on the phytochemical and pharmacological investigations upon *W. nutans*.

**Figure 1:**
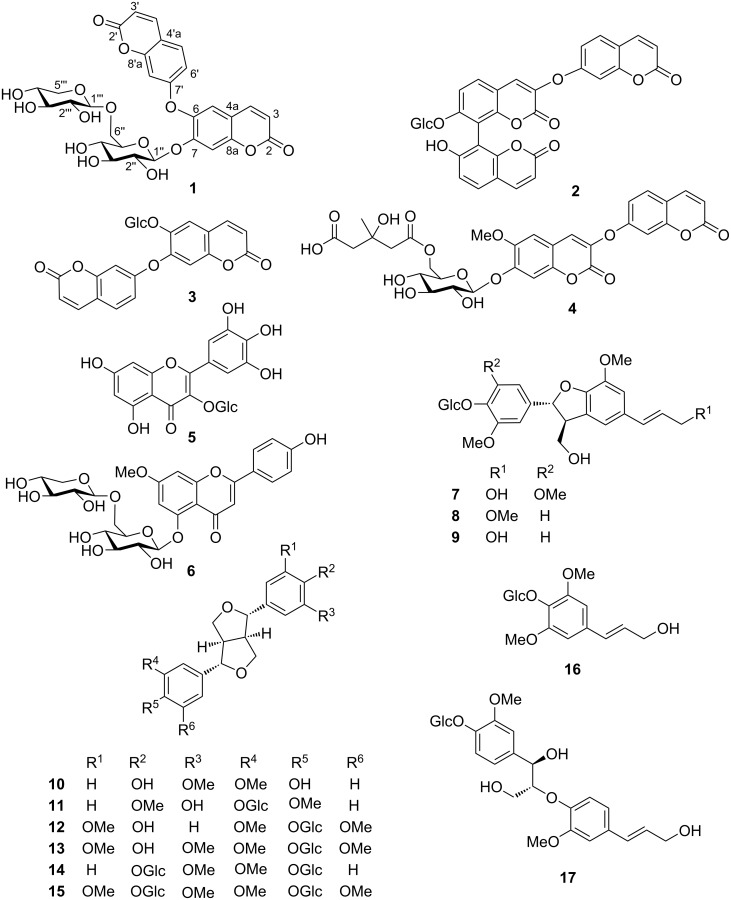
Chemical structures of compounds **1**–**17** from *W. nutans*.

Coumarins (2*H*-1-benzopyran-2-one) are a large quantity of phenolic substances found in plants and microorganisms [5]. These naturally occurring coumarins were well documented due to their diverse chemical structures and promising biological properties, such as anticancer, antitubercular, anti-inflammatory, anticoagulant, antibacterial, and neuroprotective effects [6]. As part of a continuing study of our group targeting at the identification of bioactive natural products from the medicinal plants and endophytes [7–8], the chemical constituents of the stems and roots of *W. nutans* were investigated. This work resulted into the isolation and identification of a new bis-coumarin glucoside **1**, together with three known bis- and tricoumarin glucosides **2**–**4**, two flavonoid glycosides **5** and **6**, and eleven lignan glucosides **7**–**17** (Figure 1). Herein, we present the isolation and structural elucidation of these natural products and their in vitro biological activities.

## Results and Discussion

Compound **1** was obtained as a yellowish, amorphous powder. Its molecular formula was determined as C_29_H_28_O_15_ based on the HRESIMS sodium adduct ion observed at *m/z* 639.1319 ([M + Na]^+^, calcd for C_29_H_28_O_15_Na^+^, 639.1320), indicating sixteen degrees of unsaturation. Its UV absorption bands at 325 and 293 nm indicated the presence of a coumarin-type chromophore. The IR spectrum of **1** demonstrated absorption bands characteristic for an hydroxy group (3266 cm^−1^), α,β-unsaturated carbonyl group (1739 and 1701 cm^−1^), and an aromatic ring (1624 and 1457 cm^−1^). The ^1^H NMR spectrum of **1** (Table 1) exhibited downfield chemical shifts corresponding to two pairs of olefinic protons with the AB coupling patterns at δ_H_ 7.70 (d, *J* = 9.5 Hz, H-4), 7.67 (d, *J* = 9.5 Hz, H-4'), 6.40 (d, *J* = 9.5 Hz, H-3), and 6.34 (d, *J* = 9.5 Hz, H-3'), a typical ABX coupling system at δ_H_ 7.46 (d, *J* = 8.5 Hz, H-5'), 7.01 (dd, *J* = 8.5, 2.4 Hz, H-6'), 7.02 (d, *J* = 2.4 Hz, H-8'), suggesting the presence of a 1,2,3-trisubstituted benzene ring, and two *meta*-coupling protons at δ_H_ 7.81 (s, H-8) and 7.40 (s, H-5), indicating a 1,3,4,6-tetrasubstituted benzene ring. Additionally, the ^1^H NMR spectrum revealed distinctive peaks for two anomeric protons at δ_H_ 5.71 (d, *J* = 7.7 Hz, H-1'') and 4.94 (d, *J* = 7.1 Hz, H-1'''), implied the co-existences of two sugar moieties. Analyses of the ^13^C NMR (Table 1) coupled with DEPT and HSQC spectra of **1** displayed 29 carbon signals assignable to two ester carbonyls (δ_C_ 161.3 and 161.1), seven sp^2^ quaternary carbons (including five *O*-bearing aromatic carbons at δ_C_ 162.2, 156.4, 154.0, 153.5, and 141.5; and two aromatic carbons at δ_C_ 114.7 and 114.3), nine methine groups (δ_C_ 115.4, 143.7, 121.4, 106.5, 115.0, 144.2, 130.3, 114.2, 105.3), nine oxygenated methine carbon atoms (δ_C_ 106.8, 103.0, 79.0, 78.9, 77.9, 75.4, 74.8, 71.4, 71.5), as well as two oxygenated methylenes resonated at δ_C_ 70.6, and 67.7, respectively. The aforementioned information suggested that compound **1** is likely a bis-coumarin glycoside [9].

**Table 1:** ^1^H and ^13^C NMR Spectroscopic Data for **1** (δ in ppm, *J* in Hz).

position	δ_C_^a^	δ_H_^a^	δ_C_^b^	δ_H_^b^

2	161.3		160.1	
3	115.4	6.40 (1H, d, 9.5)	114.11 ^c^	6.40 (1H, d, 9.5)
4	143.7	7.70 (1H, d, 9.5)	143.7	7.96 (1H, d, 9.5)
4a	114.3		113.3	
5	121.4	7.40 (1H, s)	120.7	7.58 (1H, s)
6	153.5		152.04 ^c^	
7	154.0		152.06 ^c^	
8	106.5	7.81 (1H, s)	104.5	7.41 (s, 1H)
8a	141.5		140.4	
2'	161.1		160.0	
3'	115.0	6.34 (1H, d, 9.5)	113.7	6.36 (1H, d, 9.5)
4'	144.2	7.67 (1H, d, 9.5)	141.2	8.03 (1H, d, 9.5)
4'a	114.7		114.13 ^c^	
5'	130.3	7.46 (1H, d, 8.5)	129.9	7.68 (1H, d, 8.4)
6'	114.2	7.01 (1H, dd, 8.5, 2.4)	113.6	6.94–6.96 (1H, m)
7'	162.2		160.6	
8'	105.3	7.02 (1H, d, 2.4)	104.2	6.94–6.96 (1H, m)
8'a	156.4		155.0	
Glc-C-1''	103.0	5.71 (1H, d, 7.7)	100.1	5.11 (1H, d, 7.8)
Glc-C-2''	74.8 ^c^	4.10–4.15 (1H, m)	72.9	3.04–3.09 (1H, m)
Glc-C-3''	77.9 ^c^	4.35–4.42 (1H, m)	76.5	3.25 (1H, t, 9.0)
Glc-C-4''	71.4	4.35–4.42 (1H, m)	69.2	3.15–3.18 (1H, m)
Glc-C-5''	79.0	4.10–4.15 (1H, m)	75.5	3.59–3.63 (1H, m)
Glc-C-6''	70.6	4.35–4.42 (1H, m) 4.75–4.77 (1H, m)	68.3	3.90 (1H, d, 9.7) 3.59–3.63 (1H, m)
Xyl-C-1'''	106.8	4.94 (1H, d, 7.1)	104.1	4.13 (1H, d, 7.5)
Xyl-C-2'''	75.4 ^c^	4.10–4.15 (1H, m)	73.3	2.95–2.97 (1H, m)
Xyl-C-3'''	78.9	4.30 (1H, t, 9.0)	76.6	3.04–3.09 (1H, m)
Xyl-C-4'''	71.5	4.20 (1H, t, 9.0)	69.4	3.29–3.31 (1H, m)
Xyl-C-5'''	67.7	3.65–3.68 (1H, m) 4.35–4.42 (1H, m)	65.7	3.69 (1H, dd, 5.3, 11.2) 2.95–2.97 (1H, m)

^a^Spectra were recorded in pyridine-*d*_5_ (600 MHz for ^1^H and 150 MHz for ^13^C); ^b^spectra were recorded in DMSO-*d*_6_ (800 MHz for ^1^H and 200 MHz for ^13^C); assignments were confirmed by ^1^H-^1^H COSY, HSQC, HMBC and ROESY experiments; ^c^overlapped.

**Figure 2:**
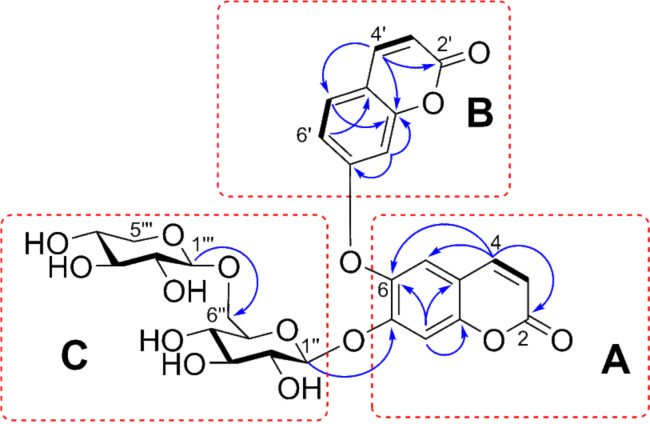
The key correlations observed in the ^1^H-^1^H COSY (bold bonds), HMBC (blue) correlations of **1** (recorded in pyridine-*d*_5_, 600 MHz).

**Figure 3:**
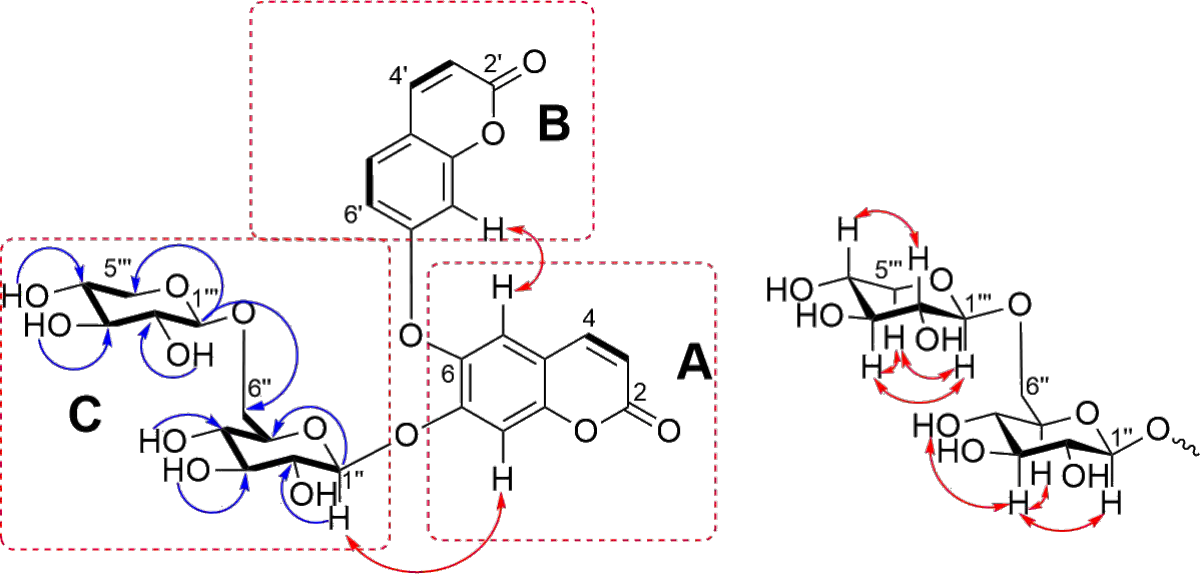
The key correlations observed in the ^1^H-^1^H COSY (bold bonds), HMBC (blue), ROESY (red) of **1** (recorded in DMSO-*d*_6_, 800 MHz).

The substructures A and B (Figure 2 and Figure 3) were elucidated based on the HMBC correlations from H-4 (δ_H_ 7.70) to C-2 (δ_C_ 161.3), C-5 (δ_C_ 121.4), and C-6 (δ_C_ 153.5), together with a ^1^H-^1^H COSY correlation between H-3 and H-4 (substructure A), while the HMBC cross-peaks from H-4' (δ_H_ 7.67) to C-2' (δ_C_ 161.1), C-5' (δ_C_ 130.3), and C-8a (δ_C_ 141.5), along with the two spin-spin systems of H-3'/H-4' and H-5'/H-6' (substructure B) depicted in the ^1^H-^1^H COSY of **1**. Although the direct evidence between substructures A and B was missing, to our delighted, the key ROESY correlation of H-5/H-8' (Figure 3 and Supporting Information File 1, Figure S34, recorded in DMSO-*d*_6_) established the C(6)–O–C(7') ether linkage. Therefore, a bis-coumarin-based aglycone was determined as shown (Figure 1), which is identical with that of daphnogitin [10].

In addition, two sugar units in substructure C (Figure 2 and Figure 3), including a β-glucopyranosyl and a β-xylopyranosyl were confirmed by the interpretation of key signals observed in the ^1^H-^1^H COSY, HSQC, HMBC, and NOSEY spectra of **1**. The β-configurations of the glucopyranosyl and xylopyranosyl units were determined by the relatively large coupling constants (*J* = 7.8 and 7.1 Hz) of their anomeric protons, respectively. The ROESY spectrum (Supporting Information File 1, Figure S34, recorded in DMSO-*d*_6_) showed correlations of H-3'' and H-1'' /H-5''/HO-4'', indicating the ᴅ-configuration of glucose, while the ROESY correlations of H-1'''/H-3'''/H-5''', H-3'''/H-5''', and H-2'''/H-4''' implied the xylose should be in ᴅ-configuration (Figure 3). The key HMBC correlations of δ_H_ 4.35 and 4.75 (Glc H-6'') with δ_C_ 106.8 (Xyl C-1''') and the reverse correlation of δ_H_ 4.94 (Xyl H-1''') with δ_C_ 170.6 (Glc C-6'') suggested the linkage of the xylopyranosyl moiety at C-6 of the glucopyranosyl unit. This deduction was further confirmed by a ROESY correlation between H-6'' and H-1'''. Subsequently, an acid hydrolysis of **1** afforded the products including daphnogitin, a ᴅ-glucose, and a ᴅ-xylose. The absolute configurations of glucopyranosyl and xylopyranosyl was further determined by HPLC analysis of the sugar derivatives (Supporting Information File 1, Figure S4). The linkage between the substructures A and C was revealed by the crucial HMBC correlation between the anomeric proton at Glc H-1'' (δ_H_ 5.71)/C-7 (δ_C_ 154.0). Therefore, the gross structure of **1** was determined as shown and the trivial name wikstronutin was given.

Sixteen known compounds were isolated and their structures were determined as 7-(β-ᴅ-glucopyranosyloxy)-7'-hydroxy-3-[(2-oxo-2*H*-1-benzopyran-7-yl)oxy]-[8,8'-bis(2*H*-1-benzopyran)]-2,2'-dione (**2**) [11], 6-(β-ᴅ-glucopyranosyloxy)-7-[(2-oxo-2*H*-1-benzopyran-7-yl)oxy]-2*H*-1-benzopyran-2-one (**3**) [9], rutarensin (**4**) [12], hirsutrin (**5**) [13], genkwanin 5-O-β-ᴅ-primeveroside (**6**) [14], (7*S*,8*R*)-5-methoxydehydrodiconiferyl alcohol 4-O-β-ᴅ-glucopyranoside (**7**) [15], 9'-methoxydehydrodiconiferyl alcohol 4-O-β-ᴅ-glucopyranoside (**8**) [16], dehydrodiconiferyl alcohol-4-O-β-ᴅ-glucopyranoside (**9**) [17], (+)-pinoresinol (**10**) [18], 4,4'-dimethoxy-3'-hydroxy-7,9':7',9-diepoxylignan-3-O-β-ᴅ-glucopyranoside (**11**) [19], (+)-medioresinol 4-O-β-ᴅ-glucopyranoside (**12**) [20], (+)-springaresinol-4''-O-β-ᴅ-monoglucopyranoside (**13**) [21], (+)-pinoresinol di-O-β-ᴅ-glucopyranoside (**14**) [22], liriodendrin (**15**) [23], syringin (**16**) [24], and citrusin A (**17**) [25] (Figure 1) by comparison to literature data. In addition, compounds **2**, **3**, **8**, and **11** were isolated for the first time from the genus *Wikstroemia*.

### Biological activity

Considered naturally occurring glycosides of phenolic metabolites usually exhibit anti-inflammatory activity in literature [26], compounds **1**−**17** were evaluated for their inhibitory activities against LPS-induced nitric oxide (NO) production in RAW 264.7 mouse macrophages. All of them showed mild inhibitory activities with inhibition rates of 10–20% at a concentration of 50 μM (Table 2). Since coumarin derivatives were reported to have antimicrobial activities [27], the antimicrobial activity of compounds **1**–**4** was also evaluated against the bacteria *Escherichia coli*, *Staphylococcus aureus subsp. aureus*, *Salmonella enterica subsp. enterica*, and *Pseudomonas aeruginosa*. However, all of them were found to be devoid of inhibitory activity (MIC >250 μg/mL).

**Table 2:** Inhibitory activities of compounds **1**−**17** on LPS-stimulated NO production.^a^

compound	inhibition (%)	compound	inhibition (%)

**1**	16.15 ± 1.10	**10**	13.85 ± 1.54
**2**	10.73 ± 1.17	**11**	18.26 ± 0.59
**3**	15.21 ± 1.51	**12**	15.90 ± 1.24
**4**	16.88 ± 1.33	**13**	19.58 ± 0.83
**5**	15.43 ± 1.22	**14**	15.42 ± 0.38
**6**	13.68 ± 1.77	**15**	15.73 ± 1.41
**7**	20.42 ± 1.06	**16**	8.16 ± 1.52
**8**	18.45 ± 1.65	**17**	7.85 ± 0.74
**9**	19.51 ± 1.11	L-NMMA^b^	52.45 ± 1.13

^a^All compounds were tested at a concentration of 50 μM.and examined in a set of triplicated experiments. ^b^Positive control.

## Conclusion

In this paper, the new bis-coumarin glucoside wikstronutin (**1**) was isolated from the stems and roots of the medicinal plant species *W. nutans*, together with three known bis- and tricoumarin glucosides **2**–**4**, two flavonoid glycosides **5** and **6**, and eleven lignan glucosides **7**–**17**. Their structures were established by extensive spectroscopic analyses, including 1D, 2D NMR spectroscopy, and HRESIMS. The relative and absolute structure of **1** was unambiguously determined based on ROESY experiments and chemical transformation. In the vitro bioassays, compounds **1**–**17** showed a mild inhibitory effect against nitric oxide (NO) production in LPS-stimulated RAW 264.7 mouse macrophages. The antibacterial activities of compounds **1**–**4** against *Escherichia coli*, *Staphylococcus aureus subsp. aureus*, *Salmonella enterica subsp. enterica*, and *Pseudomonas aeruginosa* were also tested, however, none of them showed antimicrobial activities. This is the first report of the isolation of coumarins, flavonoids, lignans and phenylpropanoid glycosides from *W. nutans*, while compounds **1**–**3**, **8**, and **11** was encountered from the genus *Wikstroemia* for the first time. Our work will enrich the chemistry and structure diversity of natural products generated by plant species from the genus *Wikstroemia*.

## Experimental

### General experimental procedures

Optical rotations were measured with a Horiba SEPA-300 polarimeter. UV spectra were recorded using a Waters UV-2401A spectrophotometer equipped with a DAD and a 1 cm path length cell. Methanolic samples were scanned from 190 to 400 nm in 1 nm steps. IR spectra were obtained using a Tensor 27 spectrophotometer with KBr pellets. 1D and 2D NMR spectra were acquired on Bruker DRX-600 and DRX-800 spectrometers with TMS as internal standard. Chemical shifts (δ) were expressed in ppm with reference to the solvent signals. Mass spectra were recorded on a VG Auto Spec-3000 instrument or an API QSTAR Pulsar 1 spectrometer. Semi-preparative HPLC was performed on an Agilent 1120 apparatus equipped with a UV detector and a Zorbax SB-C-18 (Agilent, 9.4 mm × 25 cm) column. Column chromatography was performed using silica gel (200–300 mesh and H, Qingdao Marine Chemical Co. Ltd., Qingdao, People's Republic of China), RP-18 gel (40–63 μm, Merck, Darmstadt, Germany), and MCI gel (75–150 μm; Mitsubishi Chemical Corporation, Japan). Fractions were monitored by TLC (GF254, Qingdao Marine Chemical Co. Ltd., Qingdao), and spots were visualized by heating silica gel plates sprayed with 10% H_2_SO_4_ in EtOH. All solvents were distilled prior to use.

### Plant materials

The dried stems and roots of *W*. nutans were collected in Xinzhou City of Guangxi Province, People’s Republic of China, in August 2019, and authenticated by Prof. Yongbo Xue of the Research Department of Pharmacognosy, Sun Yat-sen University. A voucher specimen (SYSUSZ-2019-X1) has been deposited in the Department of Natural Medicinal Chemistry, Sun Yat-sen University.

### Extraction and isolation

The dried root of *W*. nutans (3.5 kg) was extracted using 95% aqueous ethanol (15 L × 4 times × 2 h at room temperature) with ultrasonic assistance. The combined extracts were filtered and evaporated under reduced pressure to yield a green residue (350.6 g). The crude extract was suspended in distilled H_2_O (2 L) and successively partitioned with *n*-BuOH and EtOAc. The EtOAc extract (EE) fraction (110 g) was chromatographed over a silica gel (100–200 mesh) column (20 × 100 cm), eluted with dichloromethane/methanol (50:1 to 0:1 v/v) to afford 10 fractions (EE1-EE10). The fraction EE4 (13.3 g) was further purified by semi-preparative HPLC [phenyl column (10 × 250 mm, 5 µm particle size); mobile phase methanol/water (65:35) to yield compound **10** (20.0 mg). The fraction EE8 (13.3 g) was subjected to a HP20 gel eluting with a solvent mixture of methanol/H_2_O (20% to 100%, v/v) to produce six sub-fractions (EE8.1–8.6). The sub-fraction EE8.3 (250 mg) was further purified by semi-preparative HPLC; mobile phase methanol/water (75:35–50:60) to yield compounds **3** (20.0 mg) and **4** (18.0 mg), **7** (7.0 mg), and **17** (10.0 mg), respectively. The sub-fraction EE8.2 (310 mg) was also purified by semi-reparative HPLC; mobile phase methanol/water (65:35–30:70) to yield compounds **11** (25.0 mg), **12** (8.0 mg), and **13** (6.0 mg), respectively. The *n*-BuOH extract fraction (40.7 g) was chromatographed over a HP20 gel column (10 × 50 cm), eluted with dichloromethane/methanol (50:1 to 0:1 v/v) to afford 10 fractions (EE1–10). The *n*-BuOH extract (40.75 mg) was initially fractionated by HP20 eluting with a MeOH/H_2_O (20:80–100:0 gradient system, v/v) to obtain five fractions (B1–B5). The fraction B3 (1.4 g), B4 (5.3 g), and B5 (3.8 g) were further separated by Sephadex LH-20 CC, respectively, eluting with MeOH yielded thirteen subfractions, B3.1–3.4, B4.1–4.5, and B5.1–5.4. The sub-fraction B3.2 was subjected to silica gel CC (CH_2_Cl_2_/MeOH, 5:1–2:1, v/v) yielded compounds **15** (30.0 mg) and **8** (10.0 mg). The sub-fraction B4.2 (310 mg) was purified by semi-reparative; methanol/water (37:63 to 50:50); to yield compounds **9** (10.0 mg) and **14** (6 mg). The sub-fraction B5.4 (310 mg) was chromatographed over a HW-40 CC eluting with MeOH to obtain six fractions (B5.4.1–5.4.6), further purified by semipreparative RP-HPLC using a mobile phase of MeCN/H_2_O (20: 80–50: 50 gradient system, v/v, 3 mL/min) to afford compounds **1** (7.0 mg), **2** (15.0 mg), **5** (20.0 mg), and **6** (10.0 mg).

### Identification of new compounds

**Compound 1:** Pale yellow powder; [α]_D_^25^ –38.05 (*c* 0.1, MeOH); UV (MeOH) λ_max_ nm (log ε) 290 (0.35), 324 (0.32); IR *ν*_max_: 3266, 2925, 1739, 1701, 1624, 1457, 1261, 1020, 802, 611, 405 cm^–1^; ^1^H, ^13^C NMR data, see Table 1; ESIMS (positive ion mode): (*m/z*) 617 [M + H]^+^; HRESIMS (positive ion mode): (*m*/*z*) [M + Na]^+^, calcd for C_29_H_28_O_15_Na, 639.1320; found, 639.1309.

### Anti-inflammatory assay

The RAW 264.7 cells (2 × 10^5^ cells/well) were incubated in 96-well culture plates with or without 1 µg/mL LPS (Sigma Chemical Co., USA) for 24 h in the presence or absence of the test compounds. Aliquots of supernatants (50 µL) was then reacted with 100 µL Griess reagent (Sigma Chemical Co., USA). The absorbance was measured at 570 nm using Synergy TMHT Microplate Reader (BioTek Instruments Inc., USA). In the study, L-NMMA (Sigma Chemical Co., USA) was used as a positive control. In the remaining medium, an MTT assay was carried out to determine whether the suppressive effect was related to cell viability. The inhibitory rate of NO production = (NO level of blank control – NO level of test samples)/NO level of blank control. The percentage of NO production was evaluated by measuring the amount of nitrite concentration in the supernatants with Griess reagent as described previously [28].

### Antimicrobial assay

Compounds **1**–**4** were evaluated for their antimicrobial activities against *Escherichia coli*, *Staphylococcus aureus subsp. aureus*, *Salmonella enterica subsp. enterica*, and *Pseudomonas aeruginosa*. Antimicrobial assay was conducted by the previously described method [29]. Add the sample to be tested into the 96-well culture plate, the maximum concentration of the used compounds was 100 μM. Add bacteria liquid to each well, the final concentration is 5 × 10^5^ CFU/mL. Incubate at 37 °C for 24 h, the OD value at 595 nm was measured by a microplate reader, and the medium blank control was set in the experiment.

### Determination of absolute configurations of sugar units of **1**

Compound **1** (2 mg) was hydrolyzed with 1 N HCl (2 mL) at 90 °C for 2 h. The residue was partitioned between EtOAc and H_2_O to give the aglycone and sugar, respectively. The aqueous residue was concentrated to dryness under N_2_. The aqueous residue, ᴅ-glucose (2 mg), and ᴅ-xylose standard (2 mg) were separately dissolved in 0.5 mL anhydrous pyridine, and ʟ-cysteine methyl ester hydrochloride (2.0 mg) was then added. Each reaction mixture was heated at 60 °C for 1 h, and then 2-methylphenyl isothiocyanate (10 μL) was added to each reaction mixture and heated further for 1 h. The reaction mixture (0.5 mL) was then analyzed by HPLC and detected at 250 nm. Analytical HPLC was performed on a Welch Ultimate XB-C18 column (4.6 mm × 250 mm, 5 microm) at 35 °C with isocratic elution of 25% CH_3_CN in 0.1% H_3_PO_4_ for 40 min and subsequent washing of the column with 90% CH_3_CN at a flow rate 0.8 mL/min. Peaks at 16.54 and 19.64 min have coincided with derivatives of ᴅ-glucose and ᴅ-xylose [30].

## Supporting Information

File 1 NMR, MS, UV, IR spectra and HPLC chromatogram of derivative **1**.
