# Supplementary material for: Glycosylated coumarins, flavonoids, lignans and phenylpropanoids from Wikstroemia nutans and their biological activities
Source: Beilstein J Org Chem. 2022 Feb 16;18:200–7. doi: 10.3762/bjoc.18.23 (PMC8895025; doi:10.3762/bjoc.18.23)
Supplement: File 1 — NMR, MS, UV, IR spectra and HPLC chromatogram of derivative 1. [file Beilstein_J_Org_Chem-18-200-s001.pdf]

BEILSTEIN JOURNAL OF ORGANIC CHEMISTRY

Supporting Information

for

Glycosylated coumarins, flavonoids, lignans and phenylpropanoids from *Wikstroemia nutans* and their biological activities

Meifang Wu, Xiangdong Su, Yichuang Wu, Yuanjing Luo, Ying Guo and Yongbo Xue

Beilstein J. Org. Chem. **2022,** *18,* 200–207. doi:10.3762/bjoc.18.23

NMR, MS, UV, IR spectra and HPLC chromatogram of derivative 1

License and Terms: This is a supporting information file under the terms of the Creative Commons Attribution License (https://creativecommons.org/licenses/by/4.0). Please note that the reuse, redistribution and reproduction in particular requires that the author(s) and source are credited and that individual graphics may be subject to special legal provisions.
The license is subject to the *Beilstein Journal of Organic Chemistry* terms and conditions: (https://www.beilstein-journals.org/bjoc/terms)

Table of Contents	Page
**Figure S1:** HR-ESIMS spectrum of compound **1**.	S3
**Figure S2:** IR spectrum of compound **1**.	S3
**Figure S3:** UV spectrum of compound **1**.	S4
**Figure S4**. HPLC chromatogram of acid-hydrolyzed compound **1** and standard sugar.	S4
**Figure S5:** ^{1}H NMR spectrum of compound **1** (600 MHz, pyridine-d_5).	S5
**Figure S6.** ^{1}H NMR spectrum of compound **1** (600 MHz, pyridine-d_5) (expanded).	S5
**Figure S7.** ^{13}C NMR spectrum of compound **1** (600 MHz, pyridine-d_5).	S6
**Figure S8.** ^{13}C NMR spectrum of compound **1** (600 MHz, pyridine-d_5) (expanded).	S6
**Figure S9.** DEPT 135 spectrum of compound **1** (600 MHz, pyridine-d_5).	S7
**Figure S10.** ^{1}H,^{1}H COSY spectrum of compound **1** (600 MHz, pyridine-d_5).	S7
**Figure S11.** ^{1}H,^{1}H COSY spectrum of compound **1** (600 MHz, pyridine-d_5) (expanded).	S8
**Figure S12.** ^{1}H,^{1}H COSY spectrum of compound **1** (600 MHz, pyridine-d_5) (expanded).	S8
**Figure S13.** HSQC spectrum of compound **1** (600 MHz, pyridine-d_5).	S9
**Figure S14.** HSQC spectrum of compound **1** (600 MHz, pyridine-d_5) (expanded).	S9
**Figure S15.** HSQC spectrum of compound **1** (600 MHz, pyridine-d_5) (expanded).	S10
**Figure S16.** HMBC spectrum of compound **1** (600 MHz, pyridine-d_5).	S10
**Figure S17.** HMBC spectrum of compound **1** (600 MHz, pyridine-d_5) (expanded).	S11
**Figure S18.** HMBC spectrum of compound **1** (600 MHz, pyridine-d_5) (expanded).	S11
**Figure S19:** ^{1}H NMR spectrum of compound **1** (800 MHz, DMSO-d_6).	S12
**Figure S20:** ^{1}H NMR spectrum of compound **1** (800 MHz, DMSO-d_6) (expanded).	S12
**Figure S21:** ^{1}H NMR spectrum of compound **1** (800 MHz, DMSO-d_6) (expanded).	S13
**Figure S22:** ^{13}C NMR spectrum of compound **1** (200 MHz, DMSO-d_6).	S13
**Figure S23:** ^{13}C NMR spectrum of compound **1** (200 MHz, DMSO-d_6) (expanded).	S14
**Figure S24:** ^{1}H,^{1}H COSY spectrum of compound **1** (DMSO-d_6).	S14
**Figure S25:** ^{1}H,^{1}H COSY spectrum of compound **1** (DMSO-d_6) (expanded).	S15
**Figure S26:** ^{1}H,^{1}H COSY spectrum of compound **1** (DMSO-d_6) (expanded).	S15
**Figure S27:** HSQC spectrum of compound **1** (DMSO-d_6).	S16
**Figure S28:** HSQC spectrum of compound **1** (DMSO-d_6) (expanded).	S16
**Figure S29:** HSQC spectrum of compound **1** (DMSO-d_6) (expanded).	S17
**Figure S30:** HMBC spectrum of compound **1** (DMSO-d_6).	S17
**Figure S31:** HMBC spectrum of compound **1** (DMSO-d_6) (expanded).	S18
**Figure S32:** HMBC spectrum of compound **1** (DMSO-d_6) (expanded).	S18
**Figure S33:** ROESY spectrum of compound **1** (DMSO-d_6).	S19
**Figure S34:** ROESY spectrum of compound **1** (DMSO-d_6) (expanded).	S19

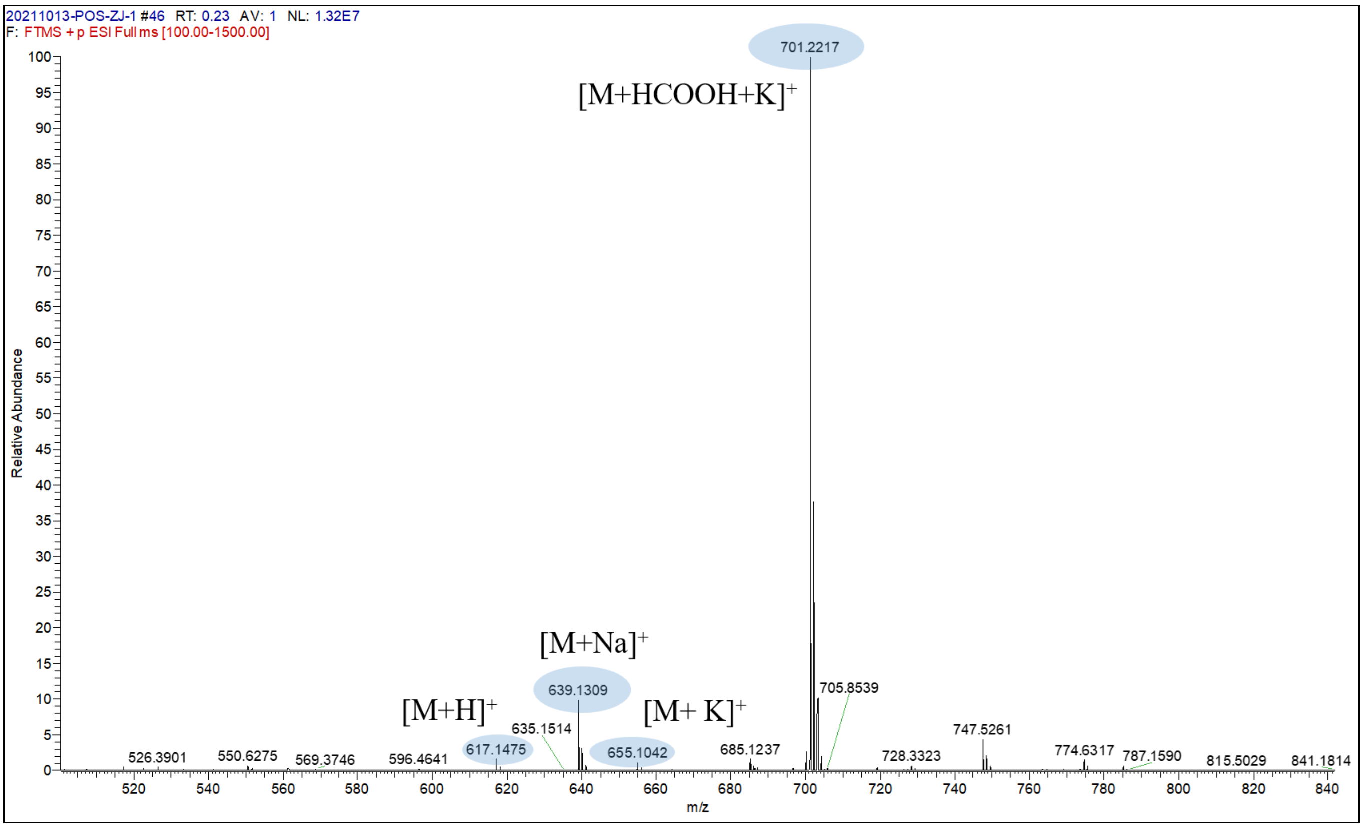

Figure S1. HR-ESIMS spectrum of compound **1**

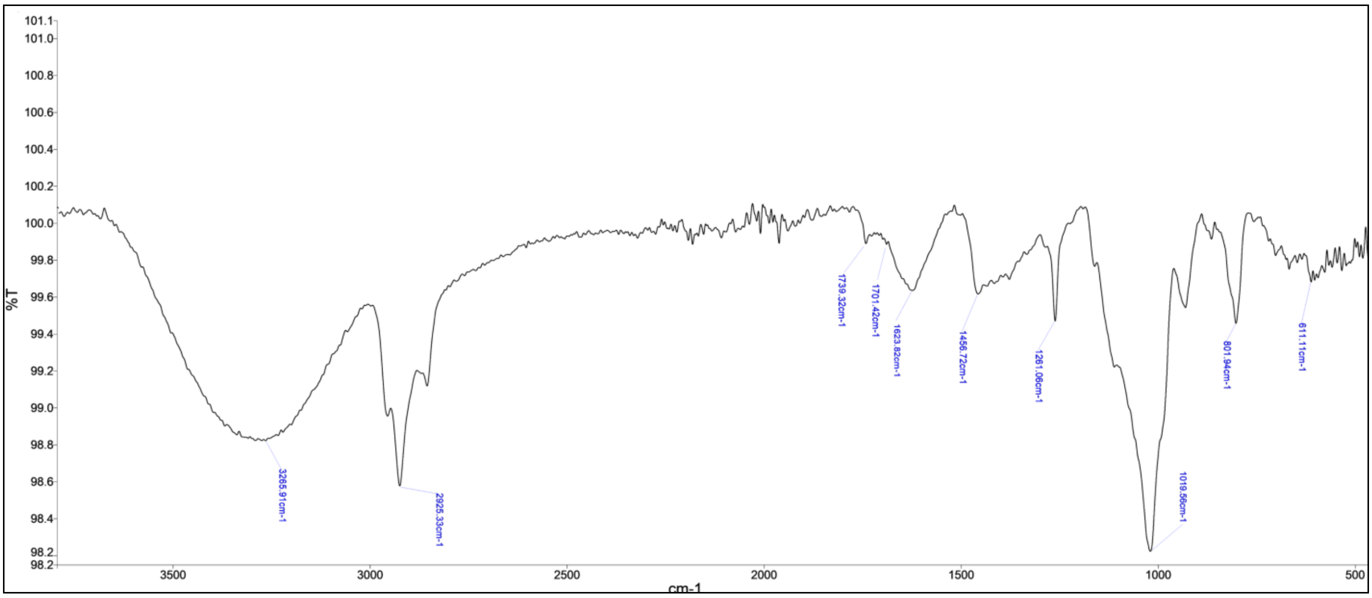

IR spectra were obtained on PerkinElmer FT-IR C106150 and Bruker Tensor 37 infrared spectrophotometers.

Figure S2. IR spectrum of compound **1** in MeOH.

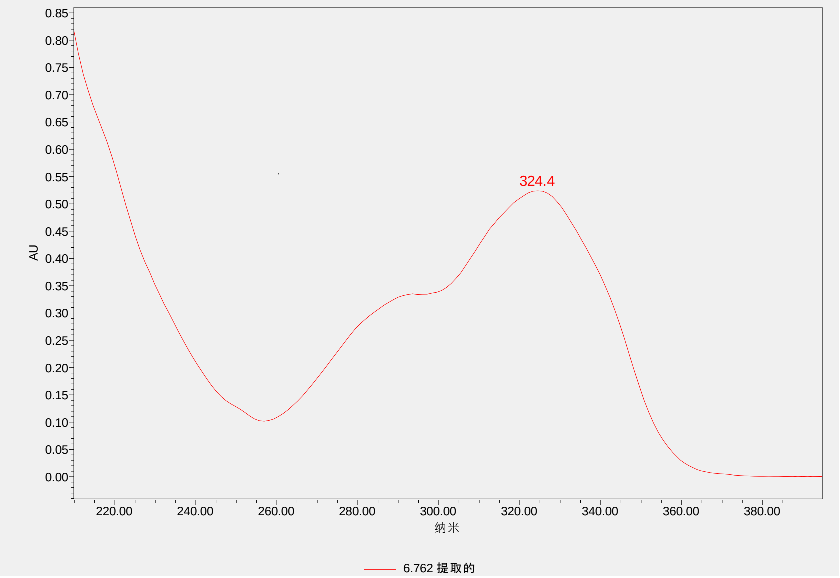

Figure S3. UV spectrum of compound **1** in MeOH.

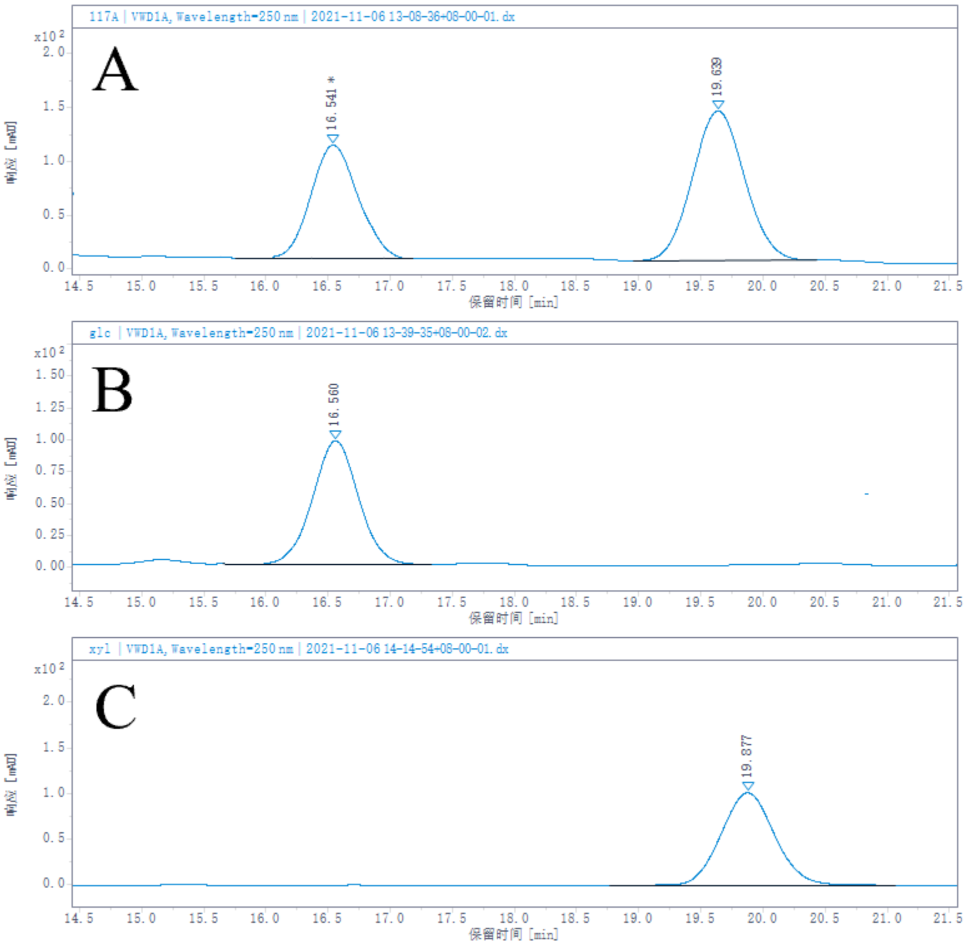

Figure S4. HPLC chromatogram of the wikstronutin (**1**) derivatives. Retention Times: (A) wikstronutin (**1**) (B) D-glucose (C) D-xylose.

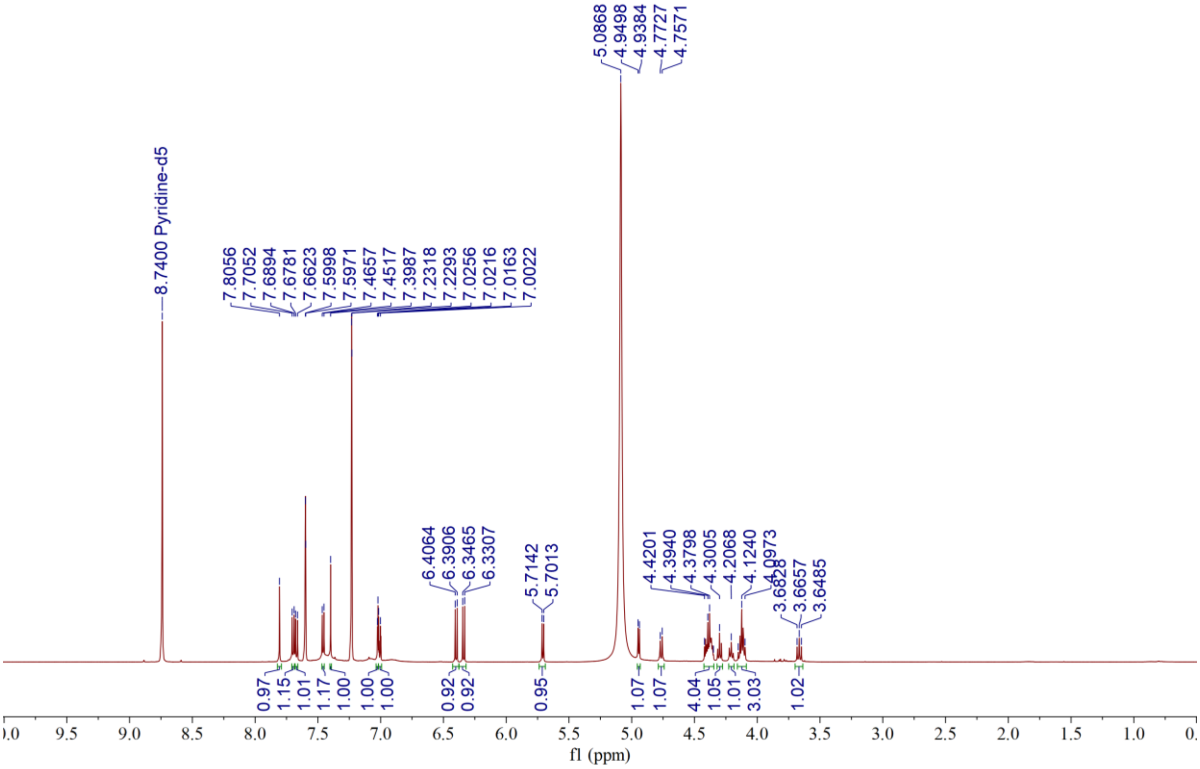

Figure S5. ^{1}H NMR spectrum of compound **1** (600 MHz, pyridine-*d*5).

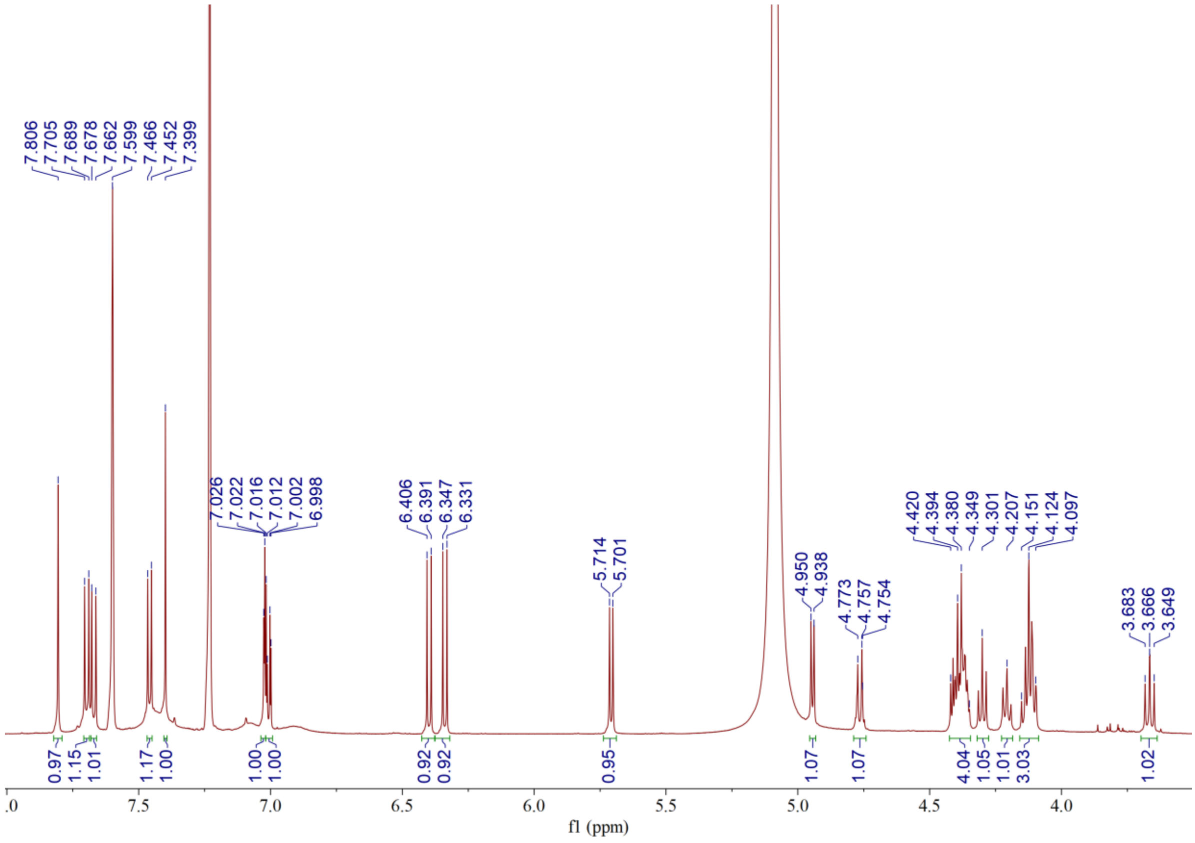

Figure S6. ^{1}H NMR spectrum of compound **1** (600 MHz, pyridine-*d*5) (expanded).

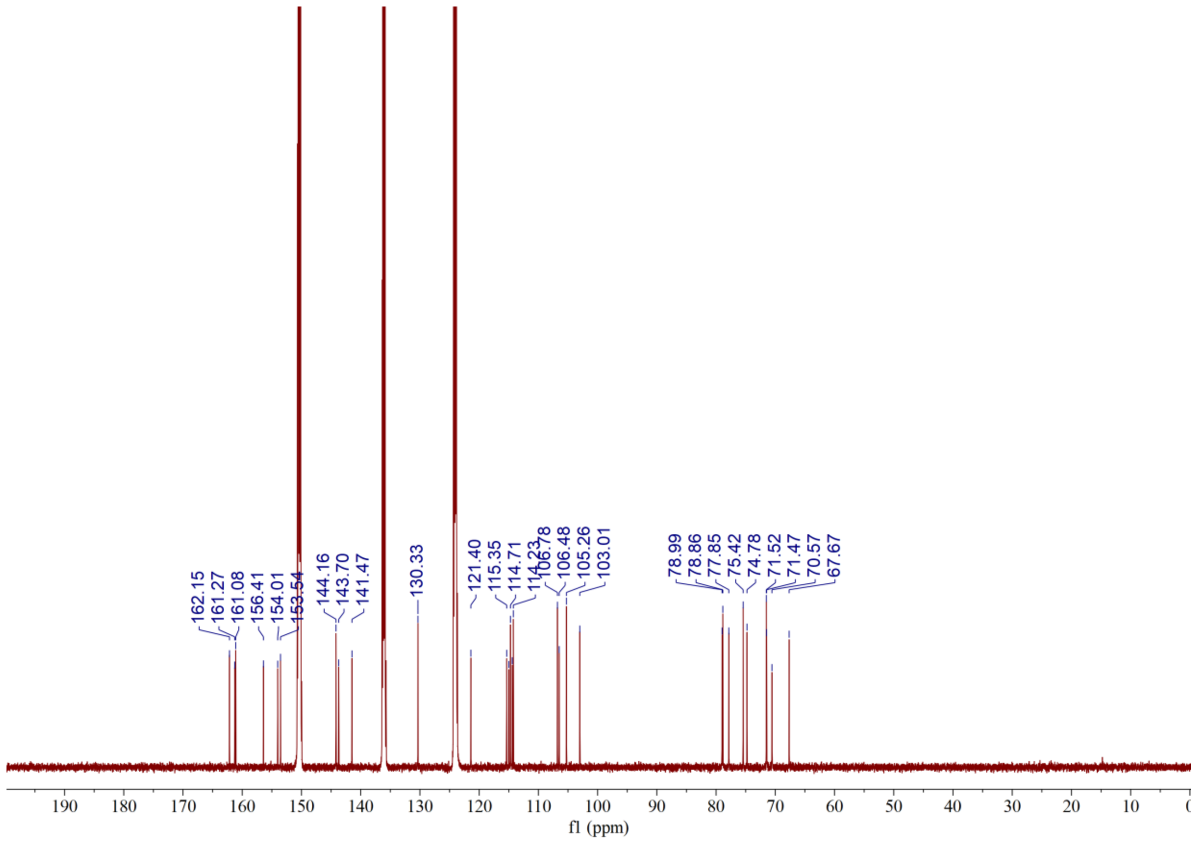

Figure S7. ^{13}C NMR spectrum of compound **1** (600 MHz, pyridine-d_5).

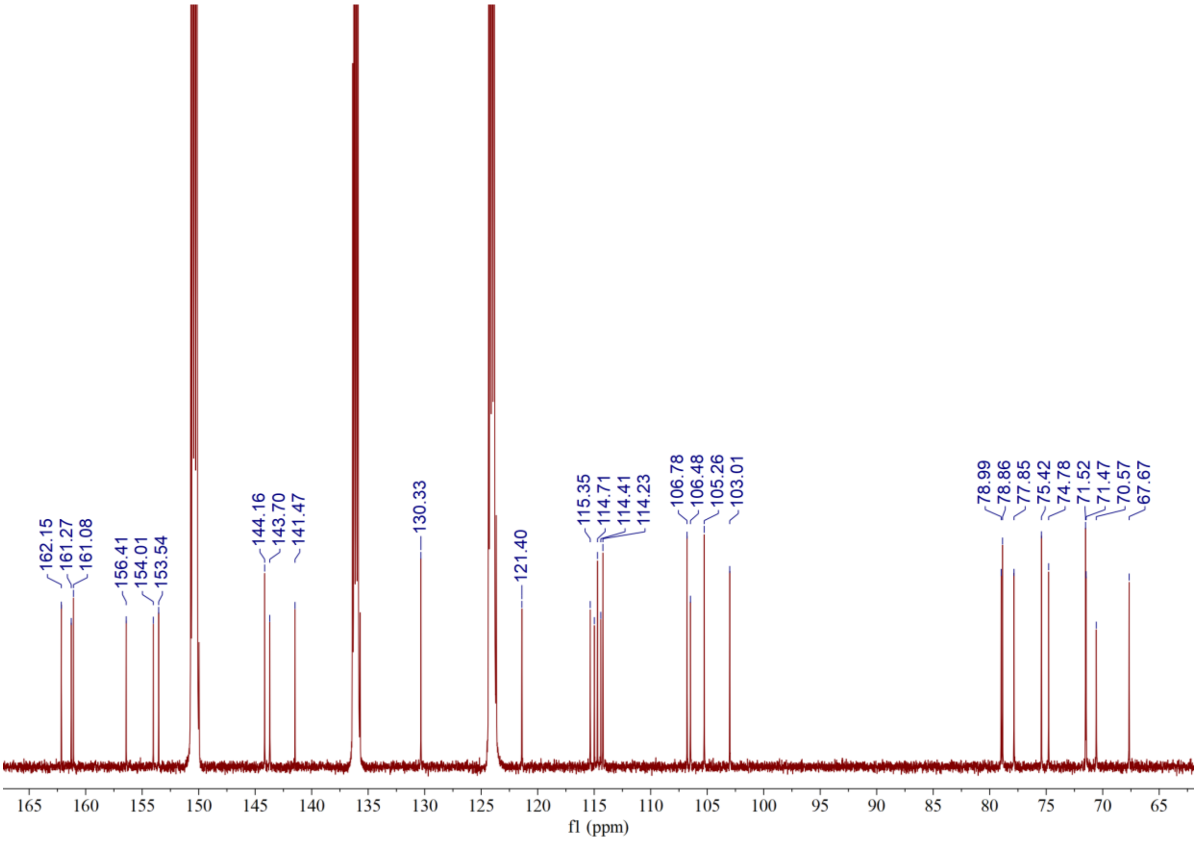

Figure S8. ^{13}C NMR spectrum of compound **1** (600 MHz, pyridine-d_5) (expanded).

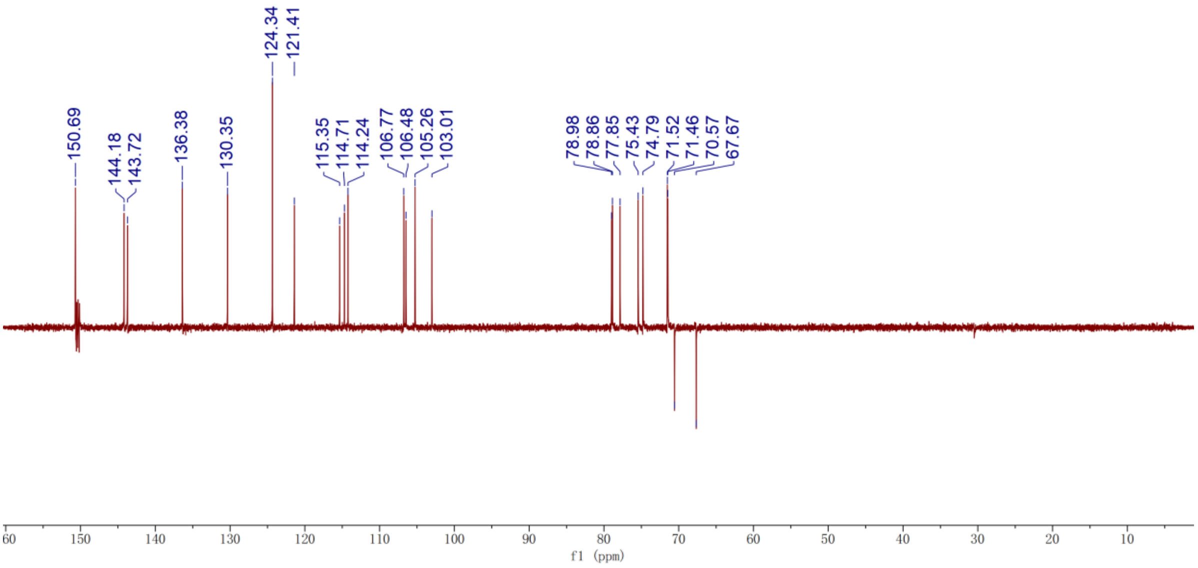

Figure S9. DEPT 135 spectrum of compound **1** (600 MHz, pyridine-d_5).

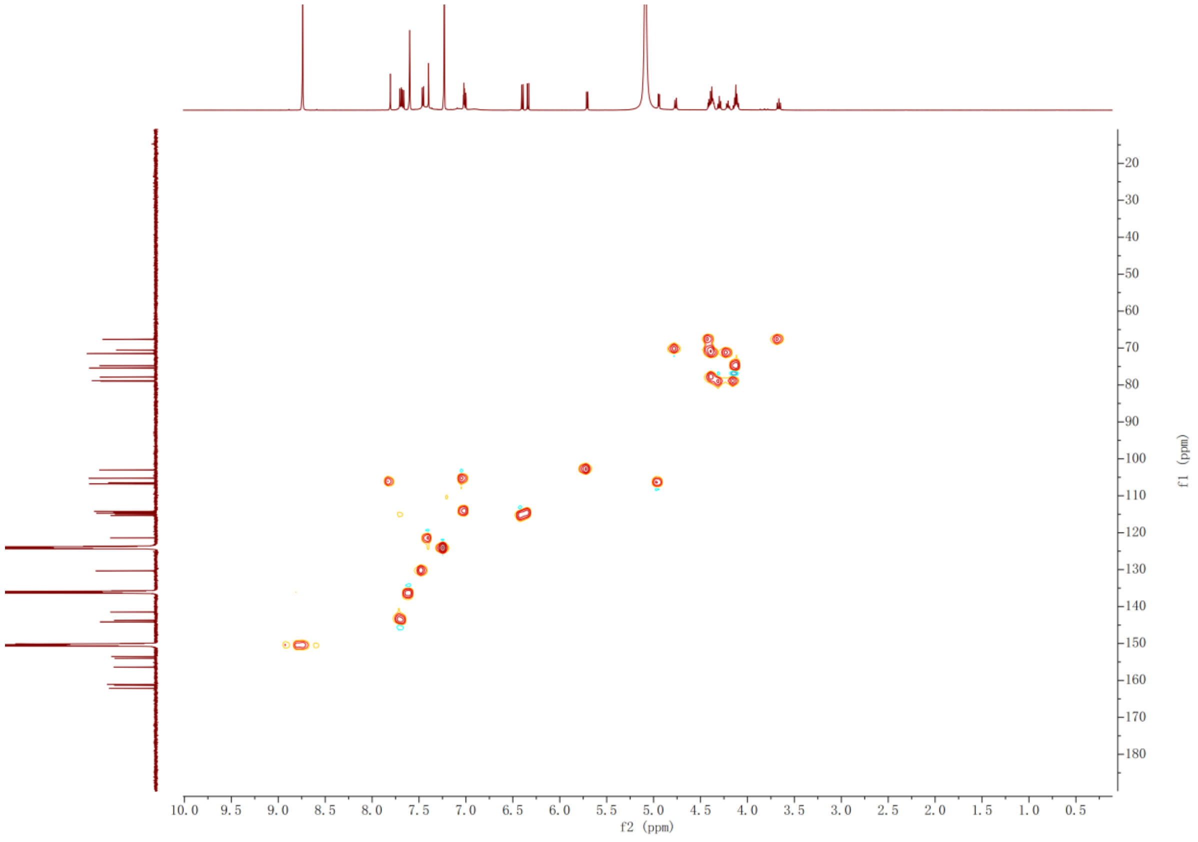

Figure S10. ^{1}H,^{1}H COSY spectrum of compound **1** (600 MHz, pyridine-d_5).

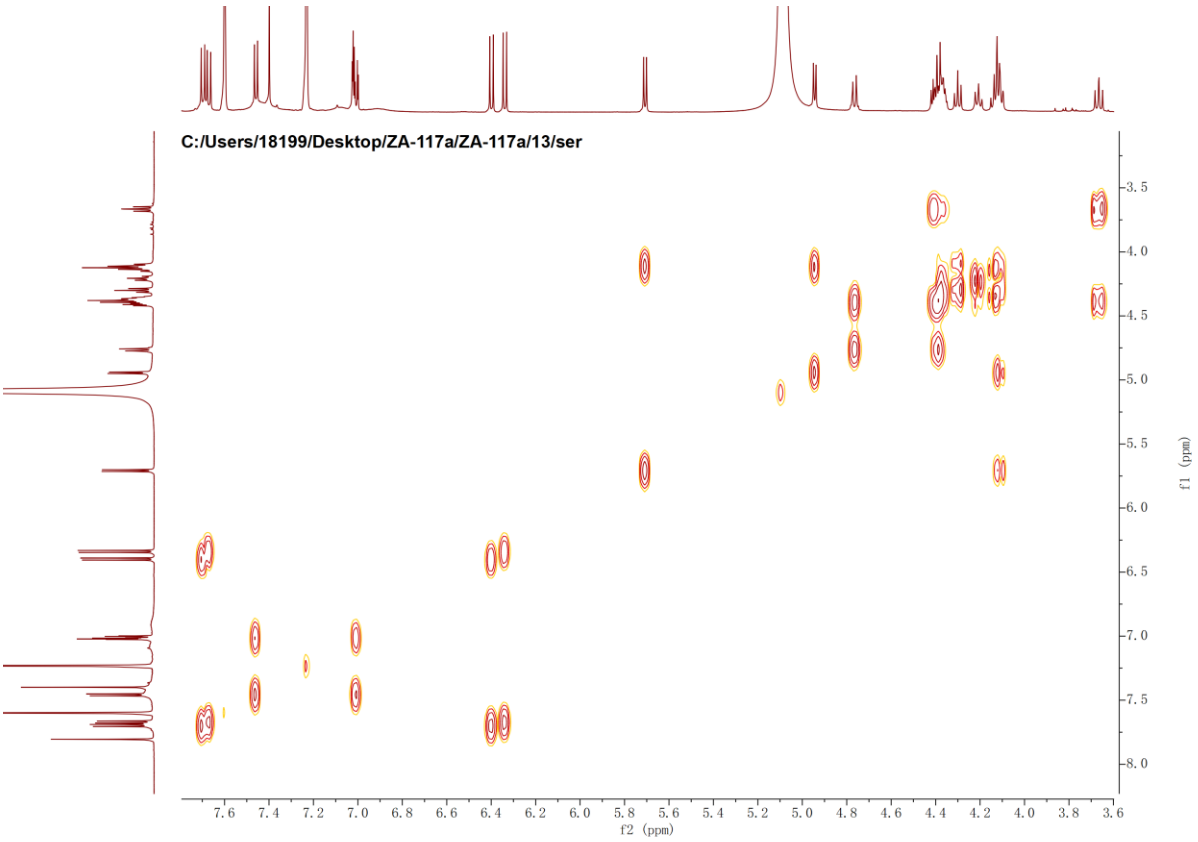

Figure S11. $^{1}H,^{1}H$ COSY spectrum of compound **1** (600 MHz, pyridine-d_5) (expanded).

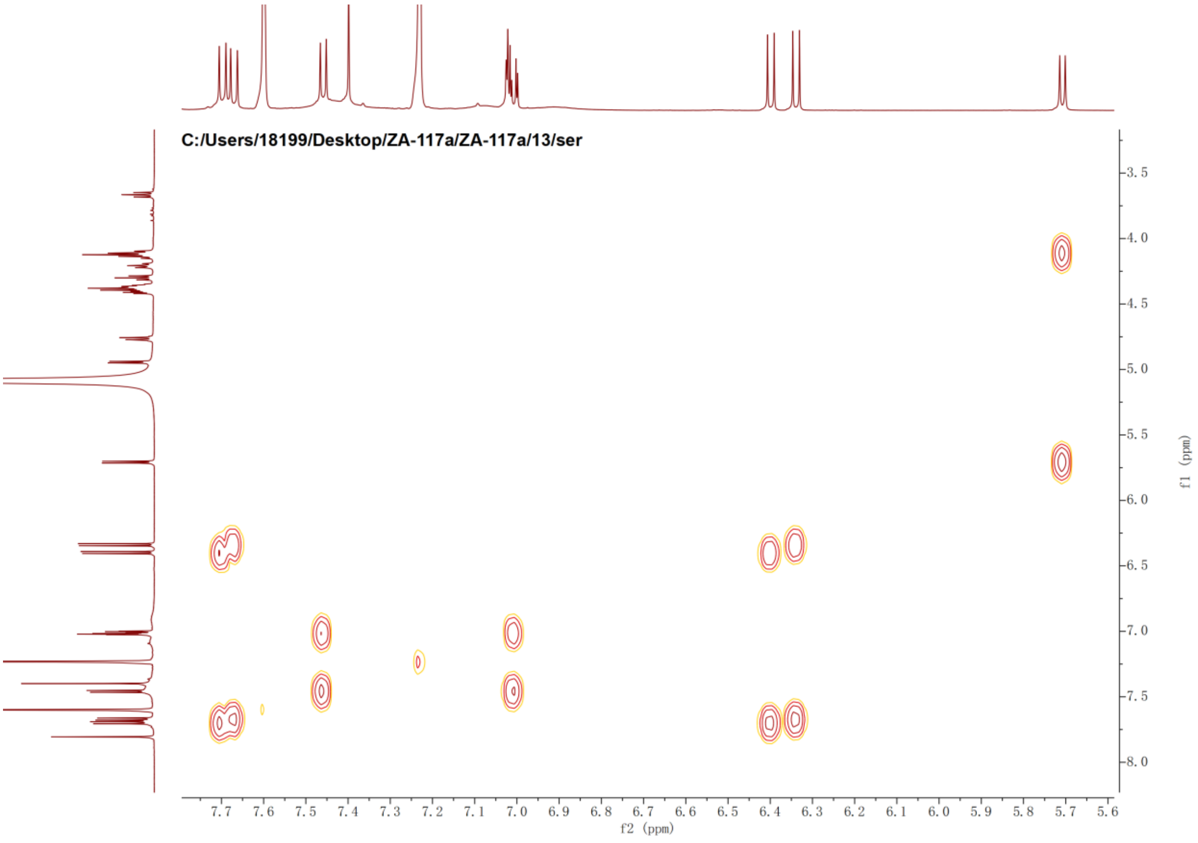

Figure S12. $^{1}H,^{1}H$ COSY spectrum of compound **1** (600 MHz, pyridine-d_5) (expanded).

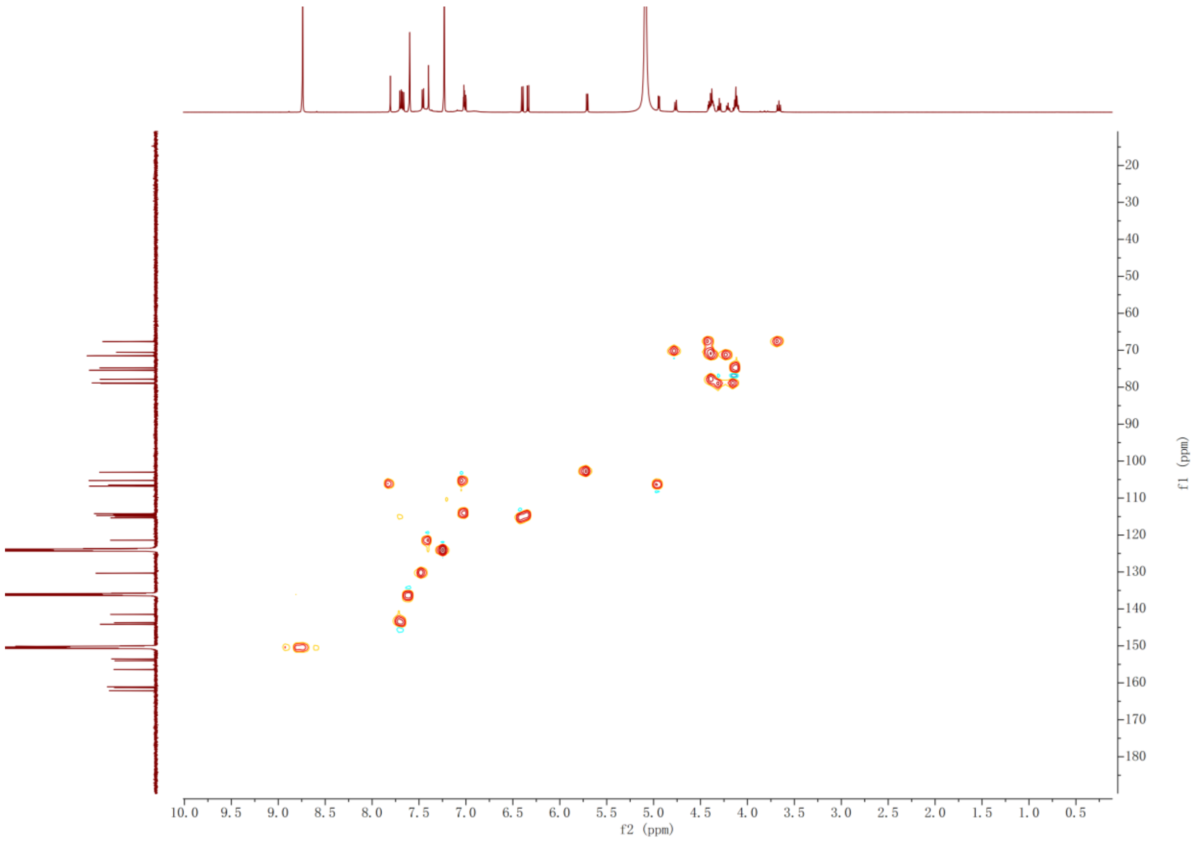

Figure S13. HSQC spectrum of compound **1** (600 MHz, pyridine-d_5).

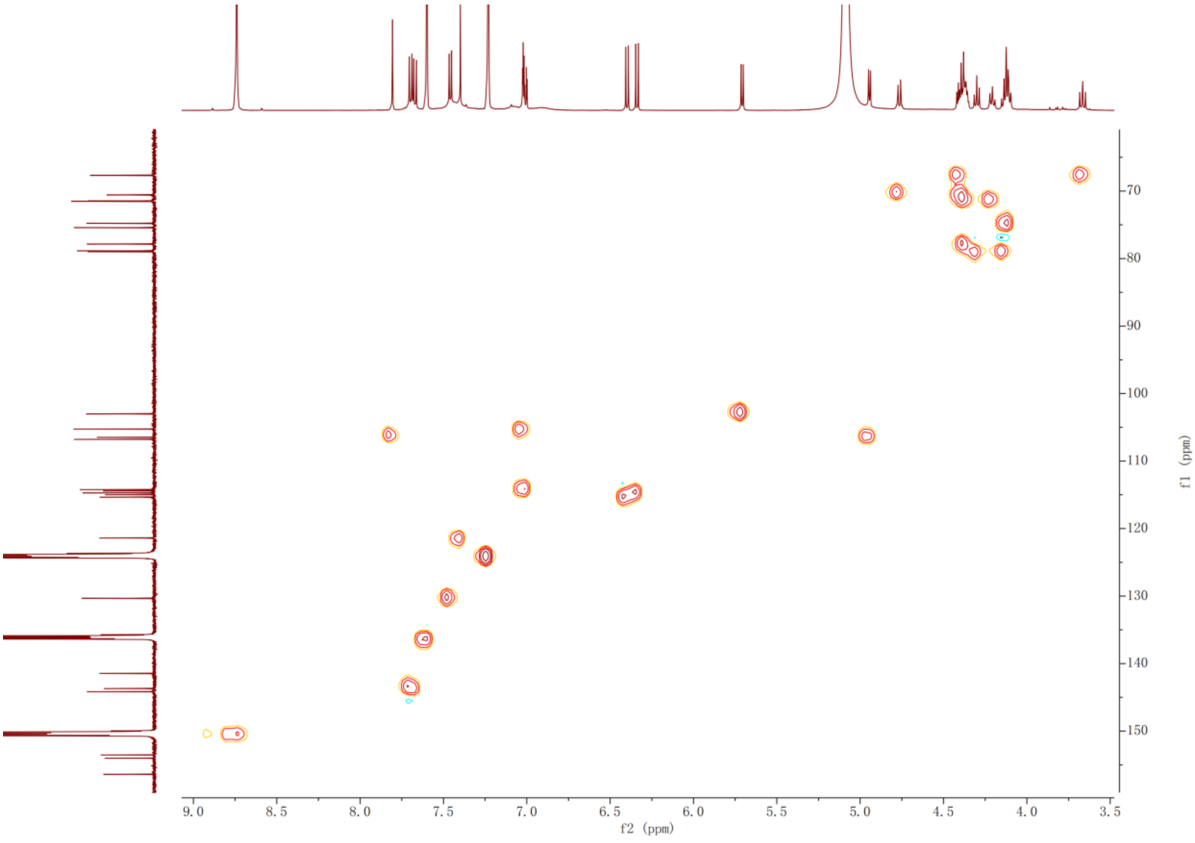

Figure S14. HSQC spectrum of compound **1** (600 MHz, pyridine-d_5) (expanded).

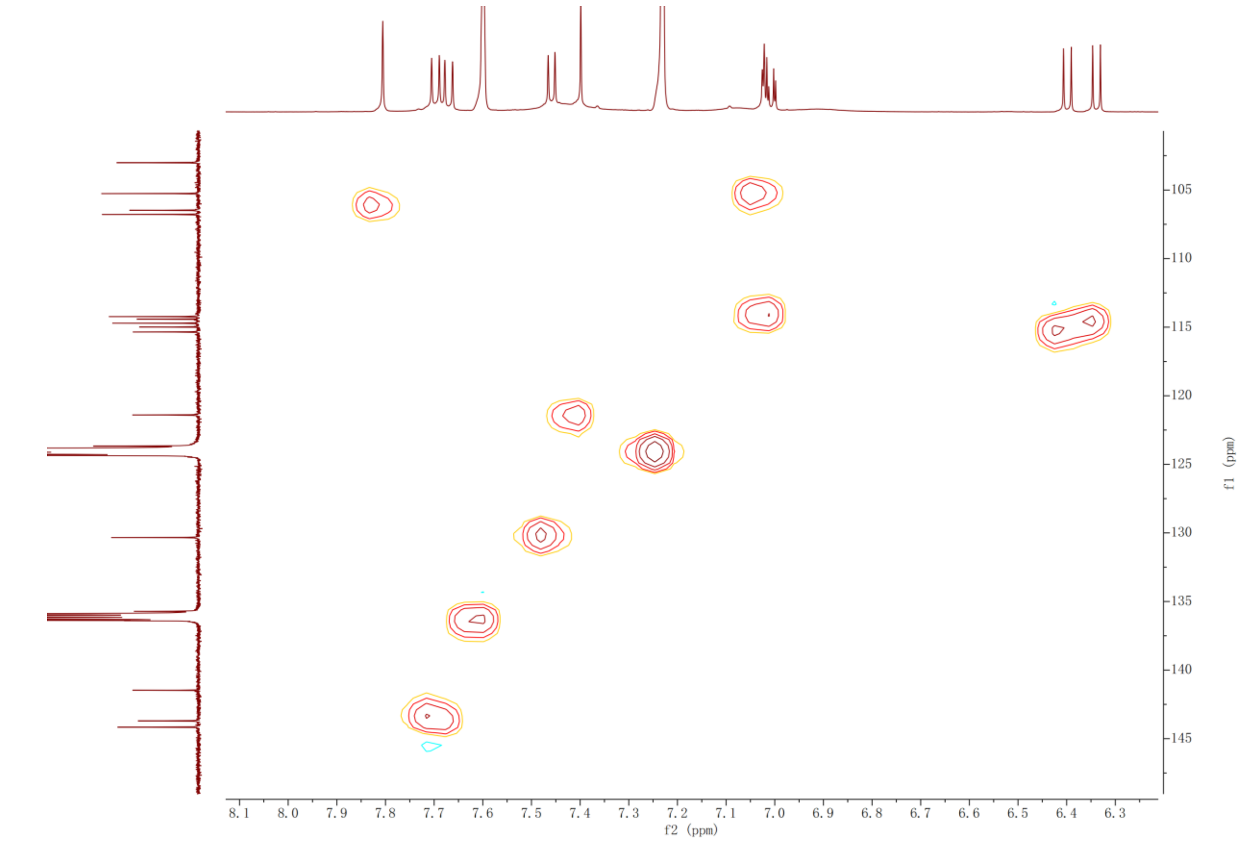

Figure S15. HSQC spectrum of compound **1** (600 MHz, pyridine-d_5) (expanded).

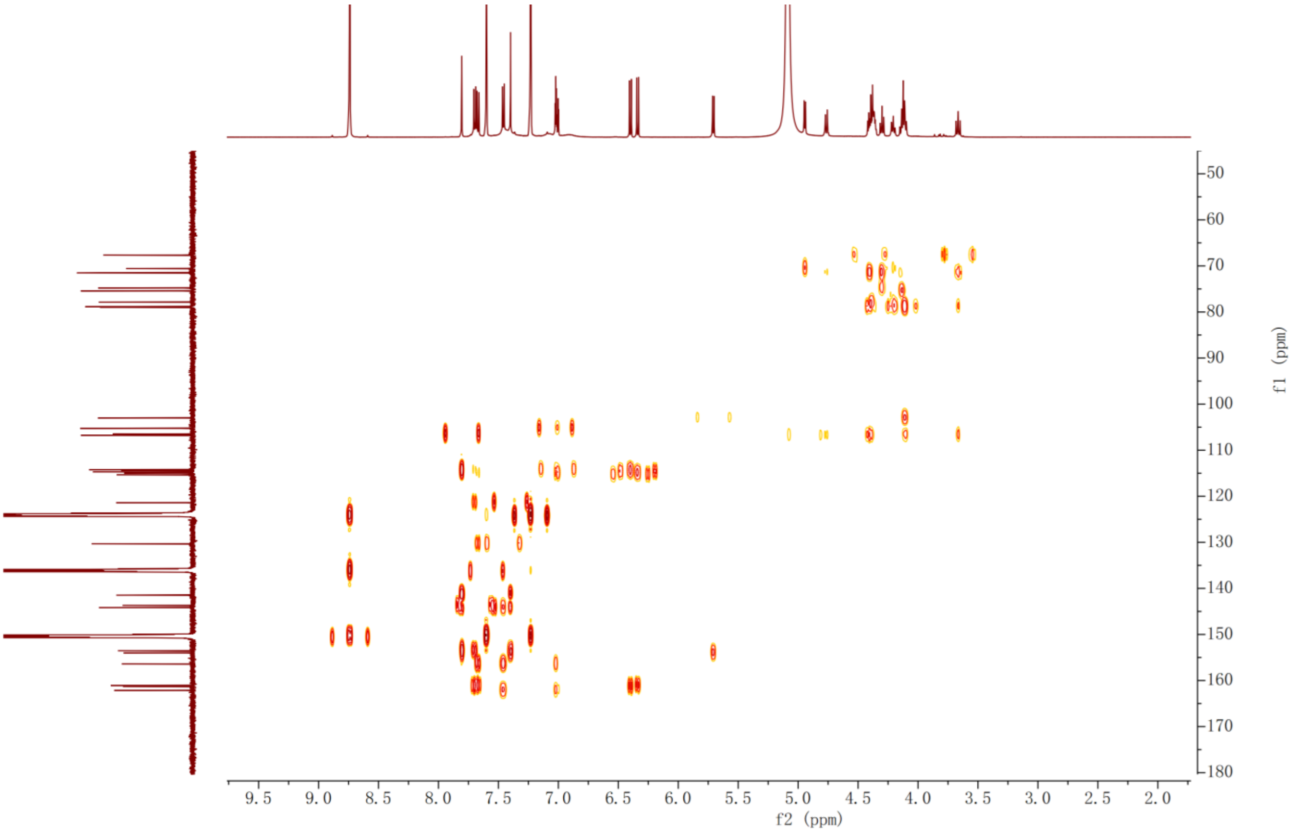

Figure S16. HMBC spectrum of compound **1** (600 MHz, pyridine-d_5).

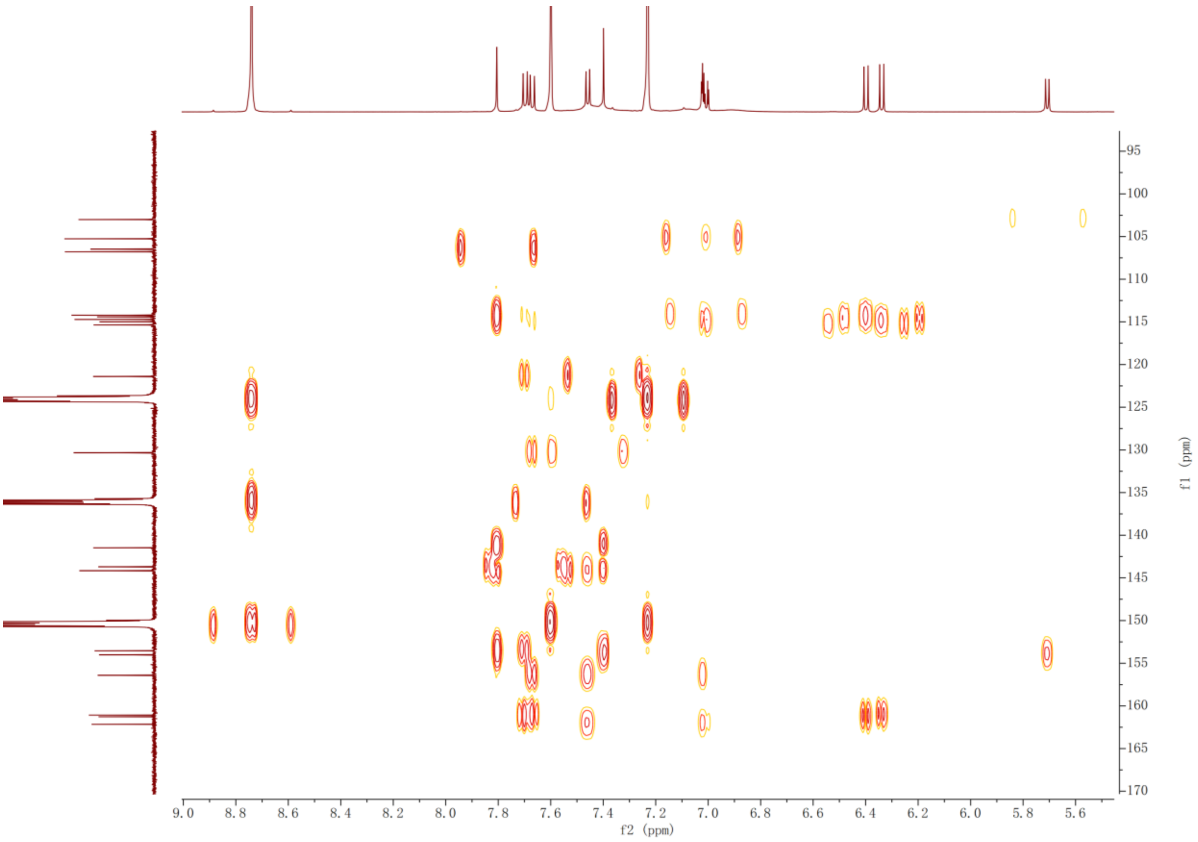

Figure S17. HMBC spectrum of compound **1** (600 MHz, pyridine-d_5) (expanded).

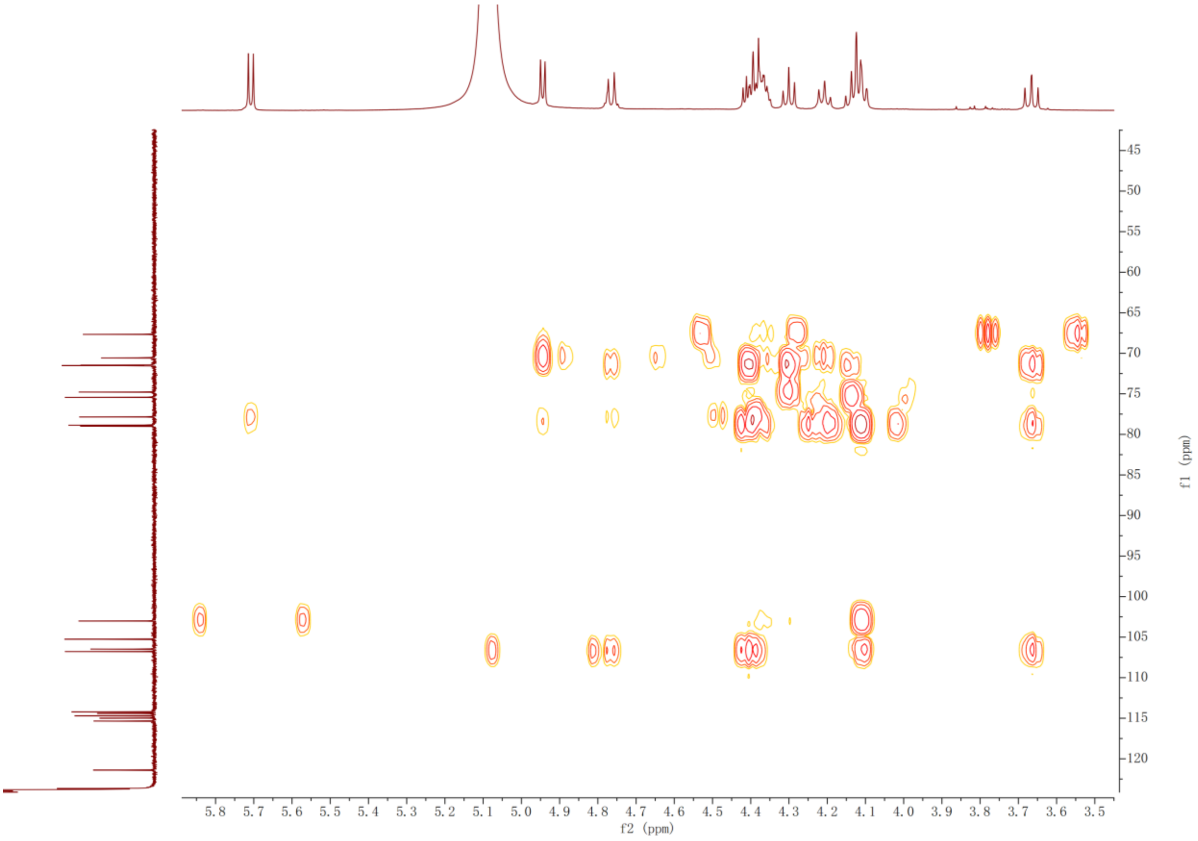

Figure S18. HMBC spectrum of compound **1** (600 MHz, pyridine-d_5) (expanded).

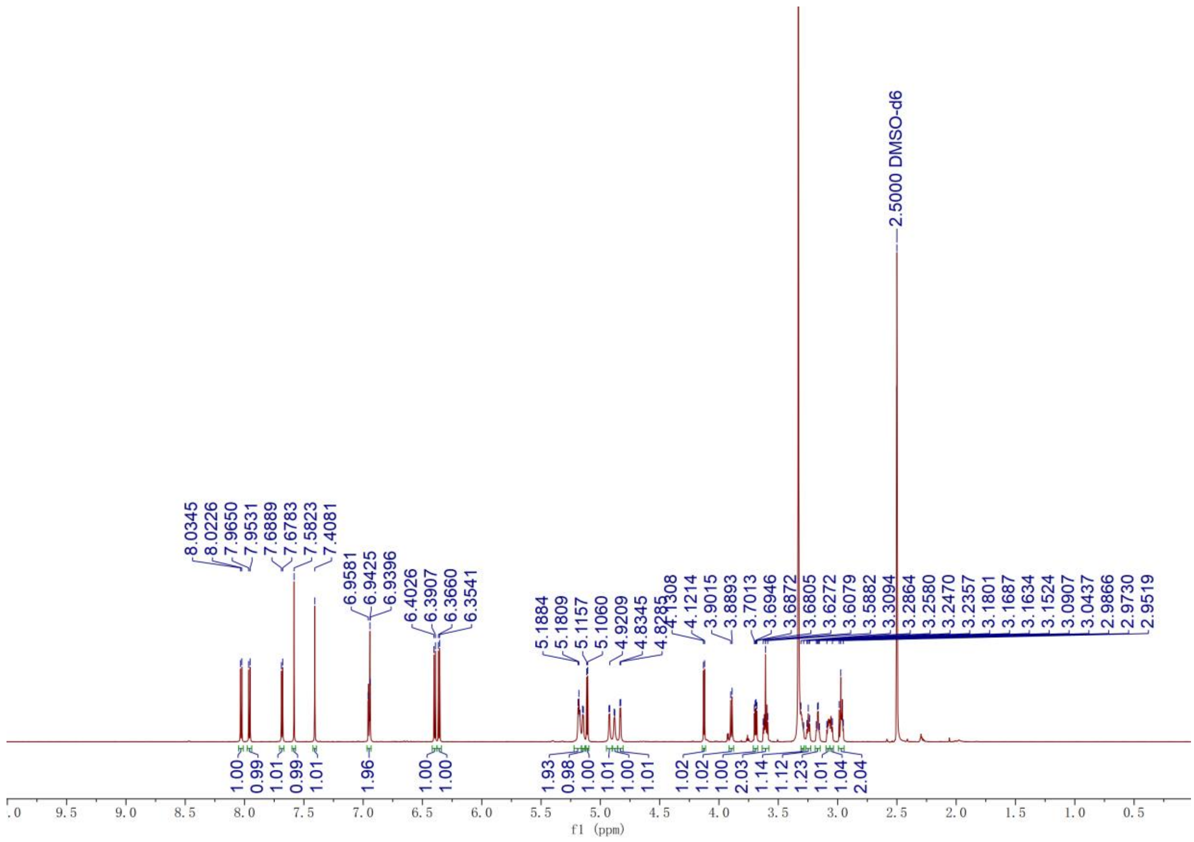

Figure S19: ^{1}H NMR spectrum of compound **1** (800 MHz, DMSO-d_6).

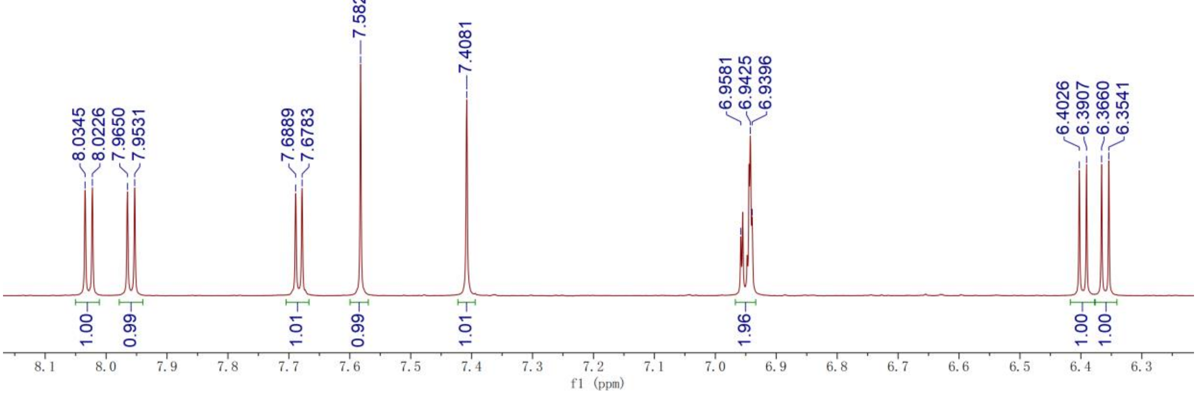

Figure S20: ^{1}H NMR spectrum of compound **1** (800 MHz, DMSO-d_6) (expanded).

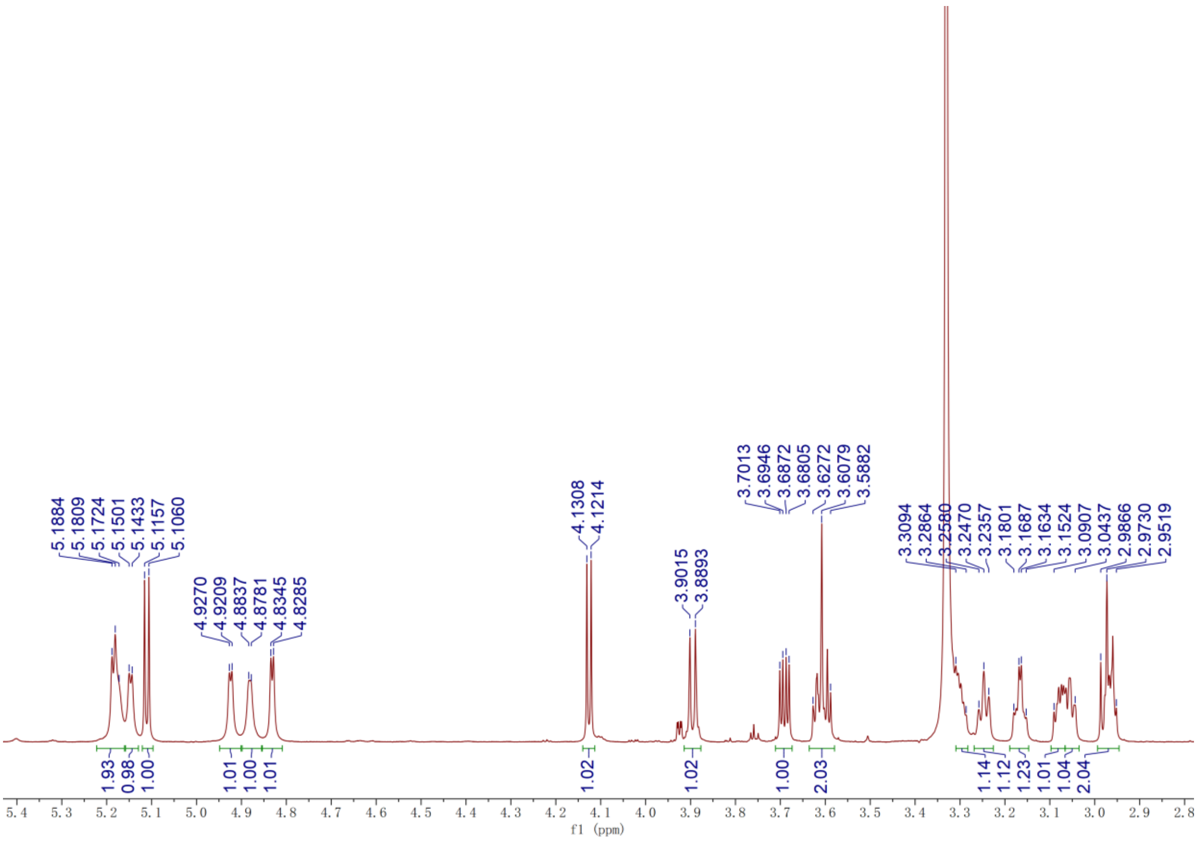

Figure S21: ^{1}H NMR spectrum of compound **1** (800 MHz, DMSO-d_6) (expanded).

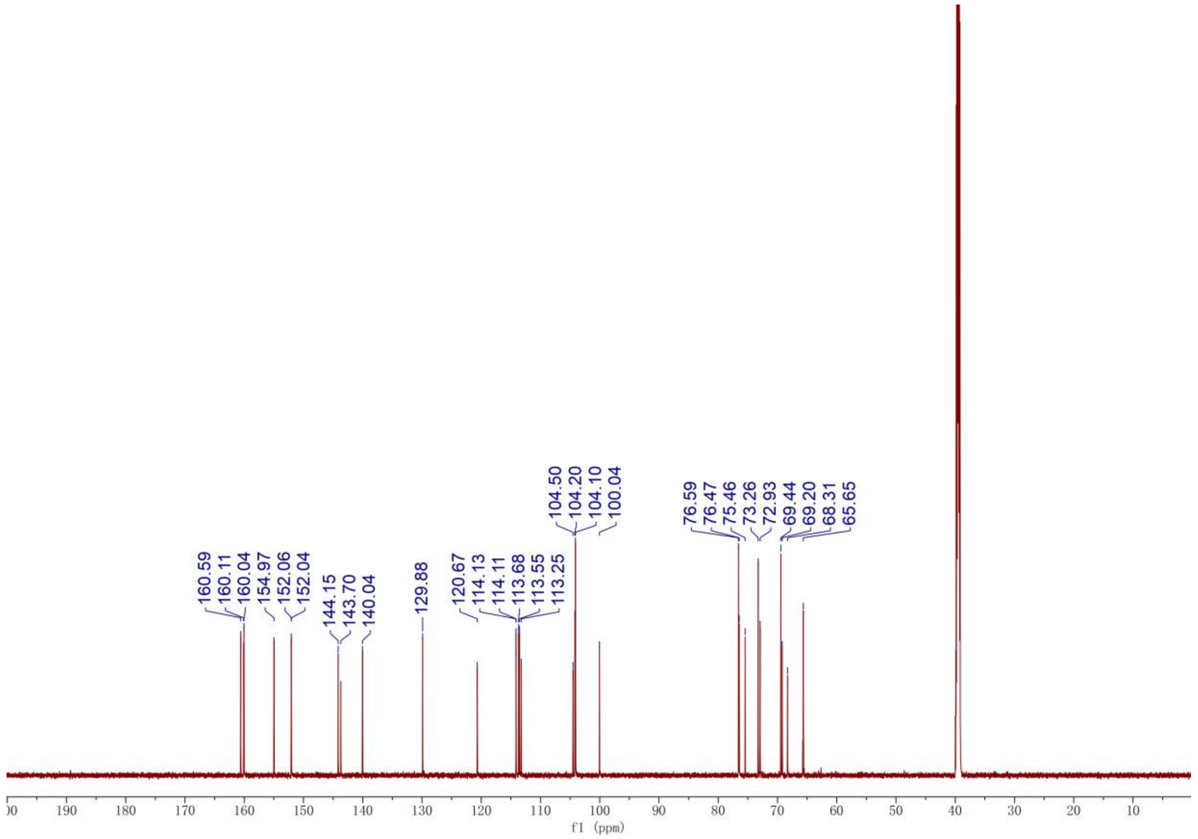

Figure S22: ^{13}C NMR spectrum of compound **1** (200 MHz, DMSO-d_6).

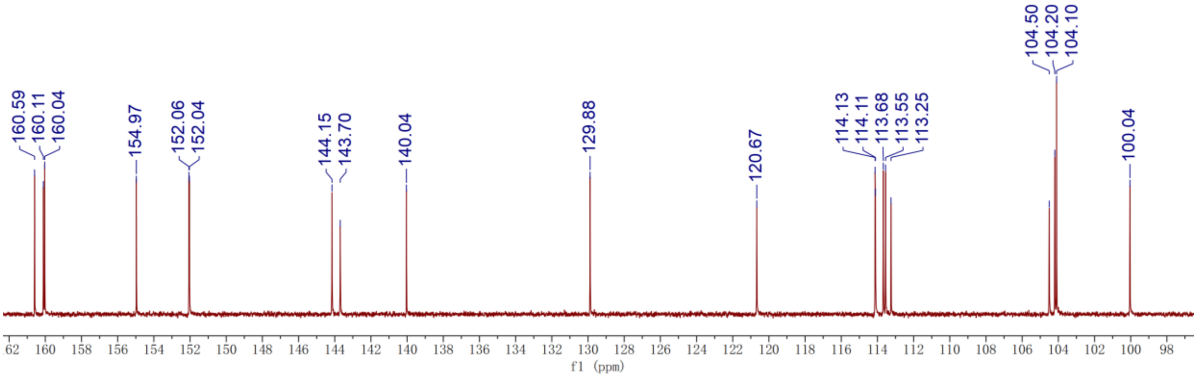

Figure S23: ^{13}C NMR spectrum of compound **1** (200 MHz, DMSO-*d*$_6$) (expanded).

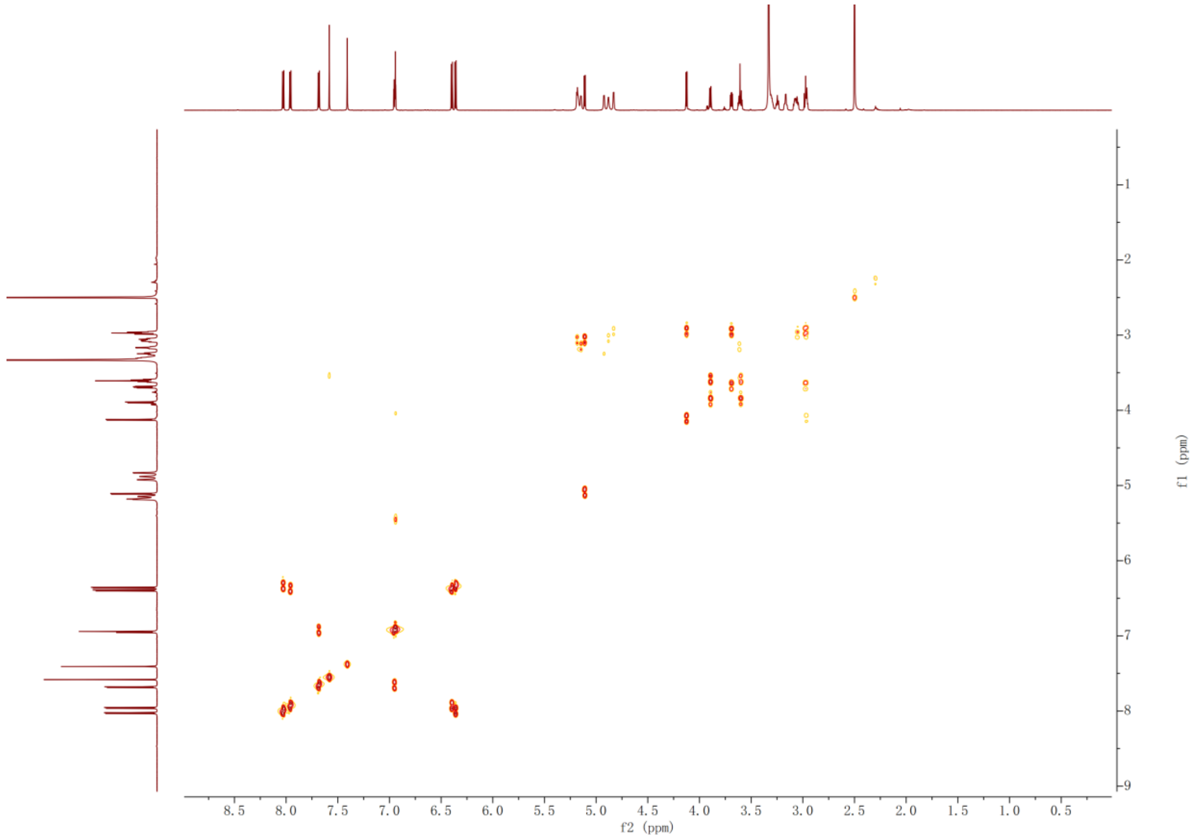

Figure S24: ^{1}H,^{1}H COSY spectrum of compound **1** (DMSO-*d*$_6$).

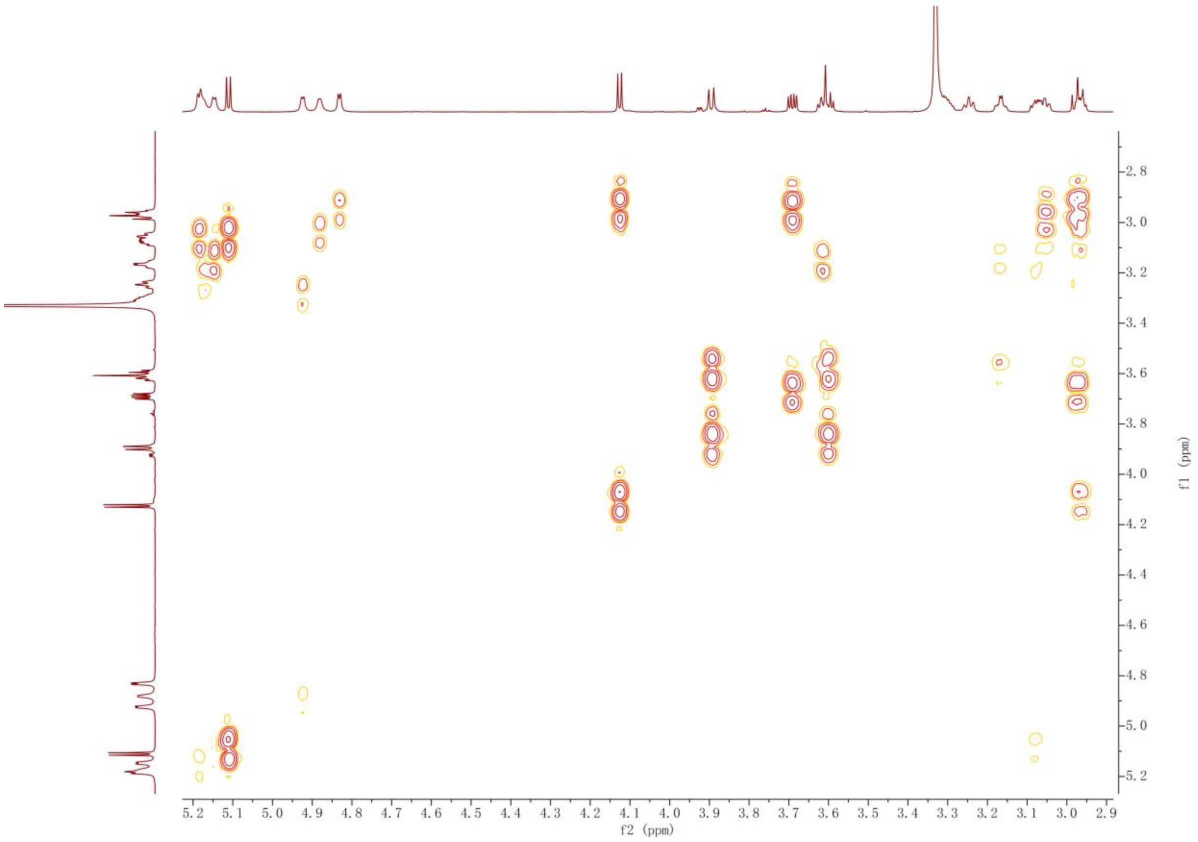

Figure S25: $^{1}H,^{1}H$ COSY spectrum of compound **1** (DMSO-d_6) (expanded).

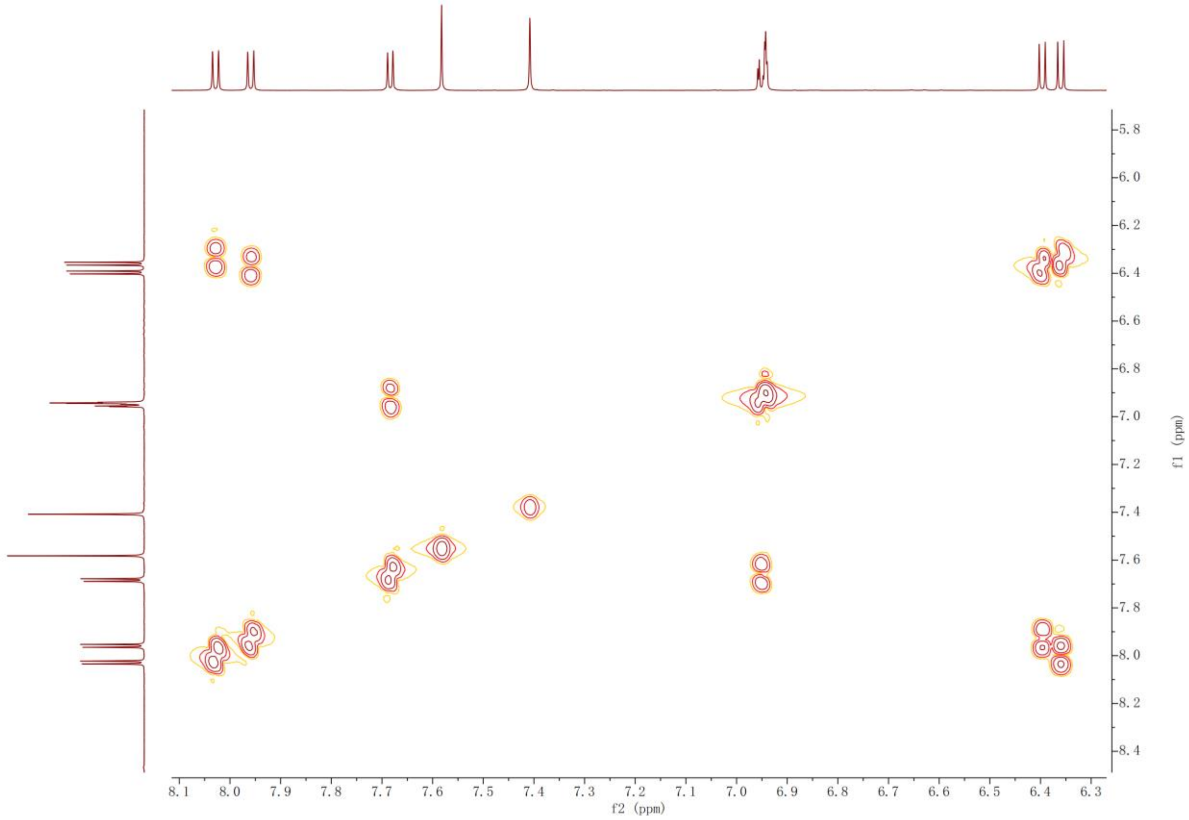

Figure S26: $^{1}H,^{1}H$ COSY spectrum of compound **1** (DMSO-d_6) (expanded).

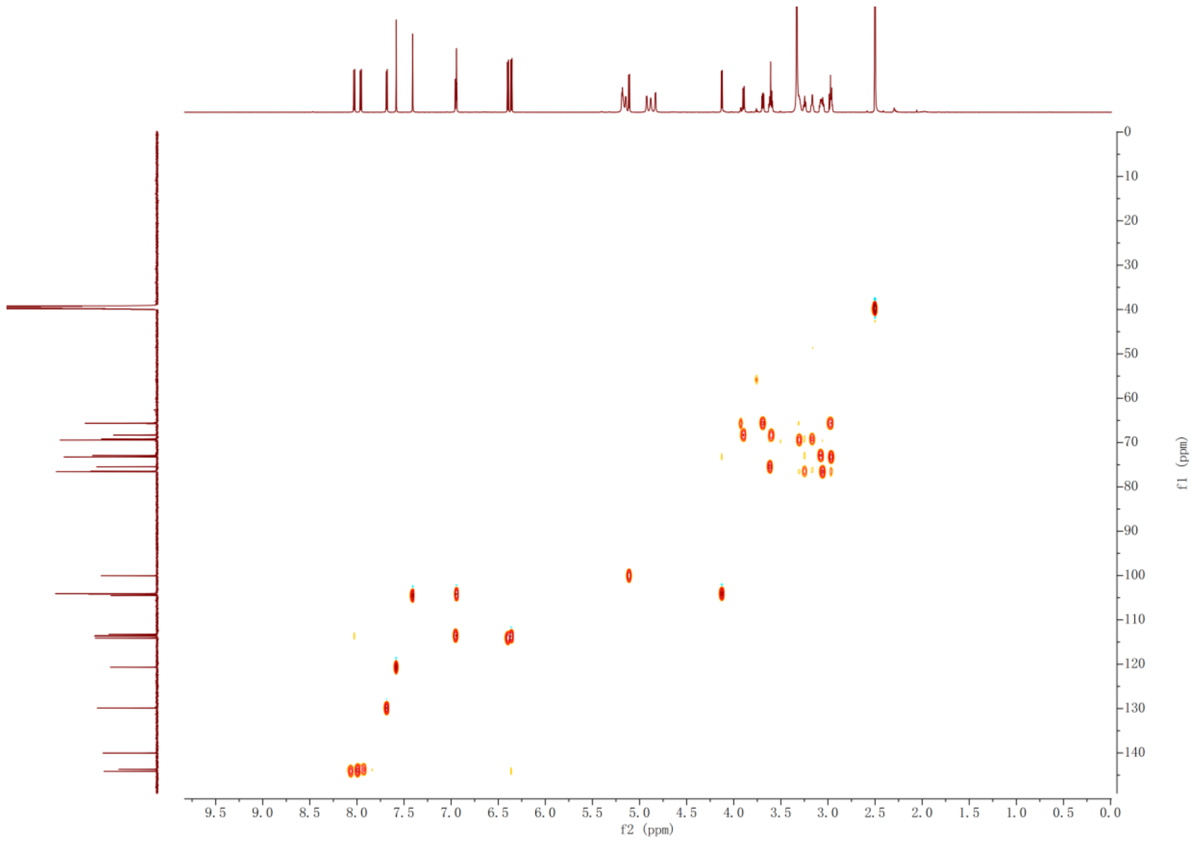

Figure S27: HSQC spectrum of compound **1** (DMSO-d_6).

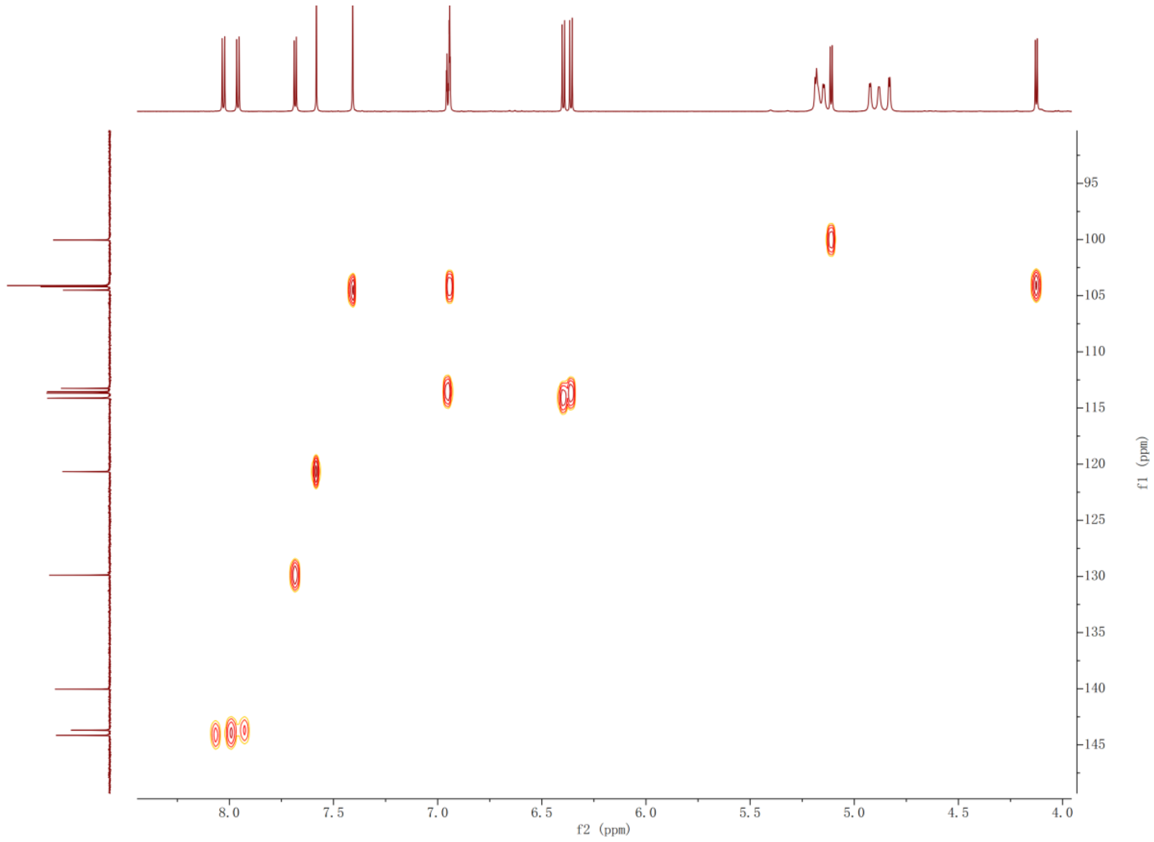

Figure S28 HSQC spectrum of compound **1** (DMSO-d_6) (expanded).

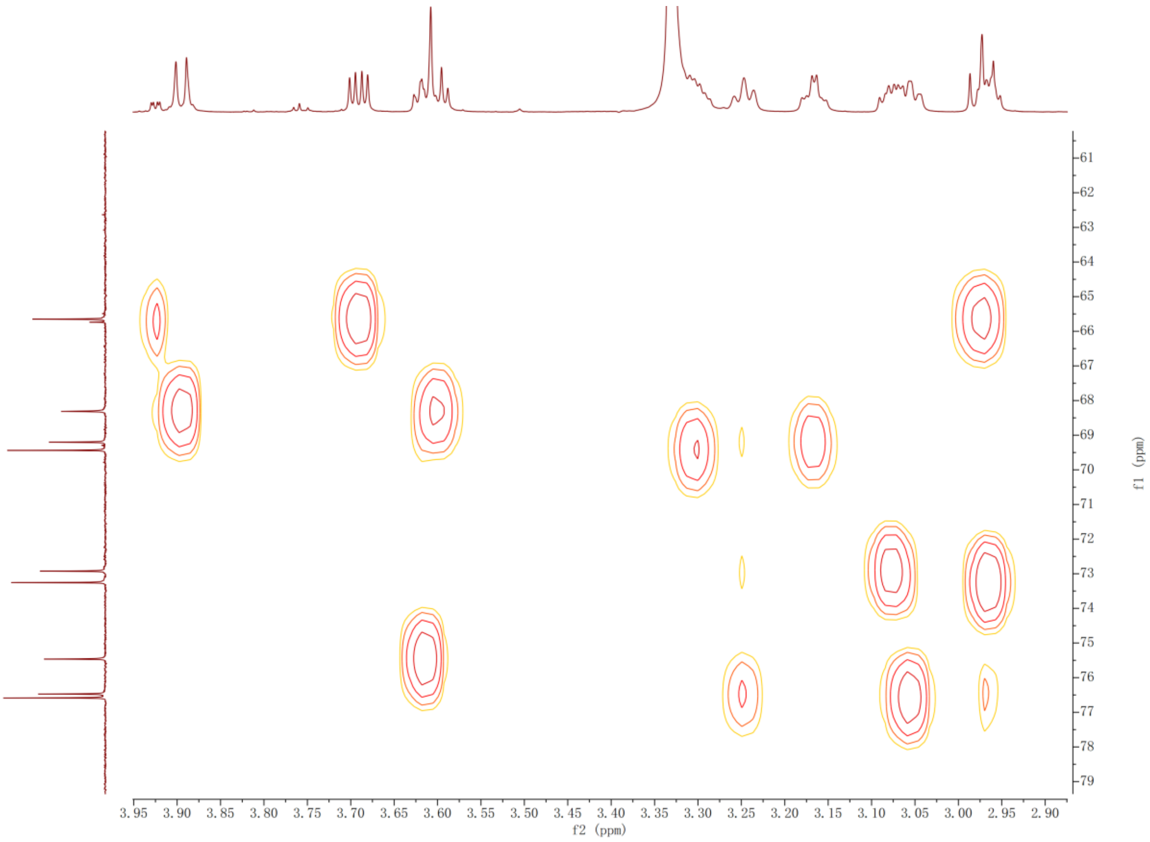

Figure S29: HSQC spectrum of compound **1** (DMSO-d_6) (expanded).

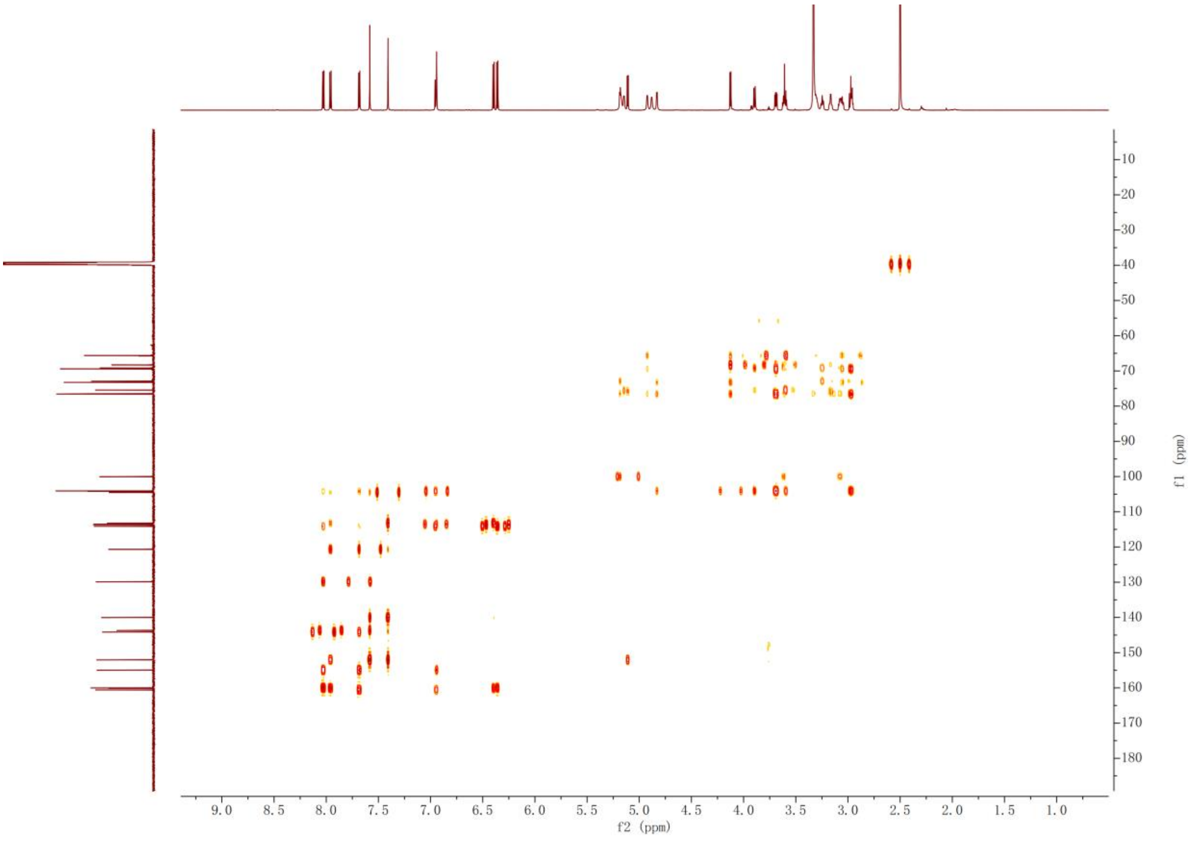

Figure S30: HMBC spectrum of compound **1** (DMSO-d_6).

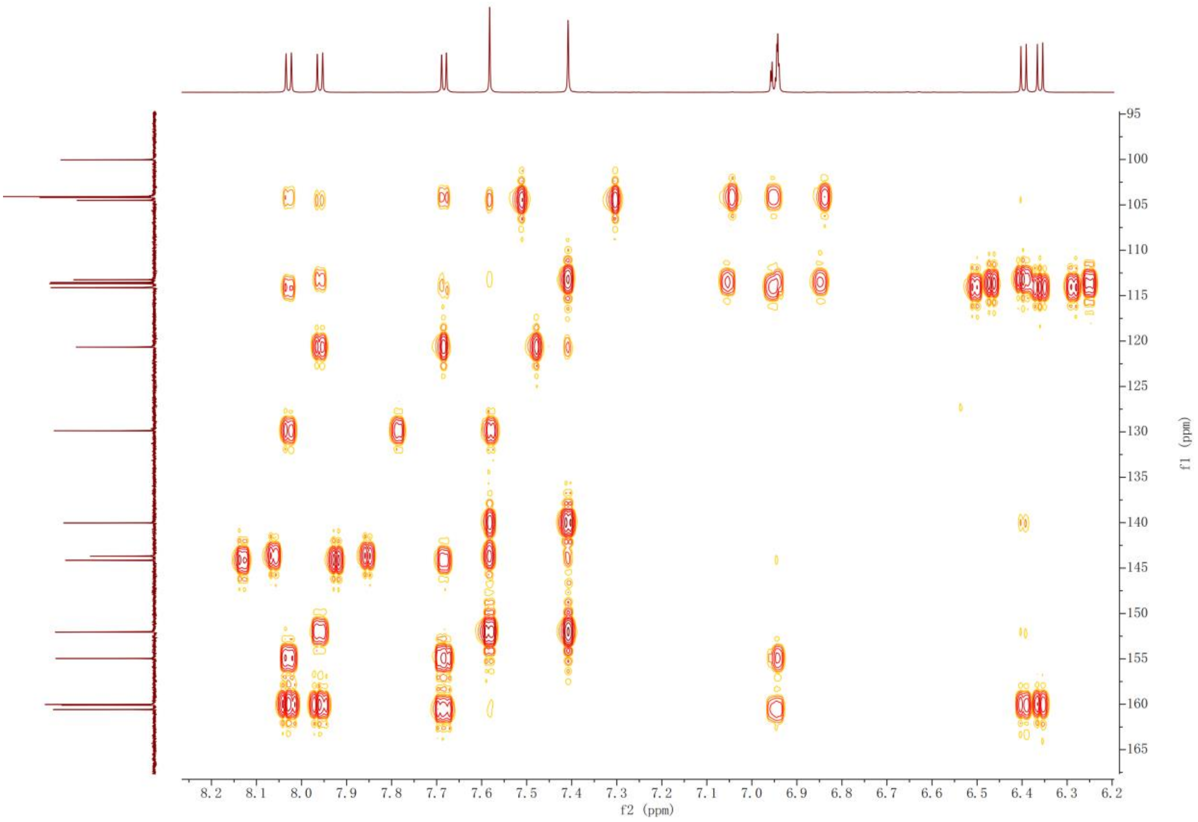

Figure S31: HMBC spectrum of compound **1** (DMSO-d_6) (expanded).

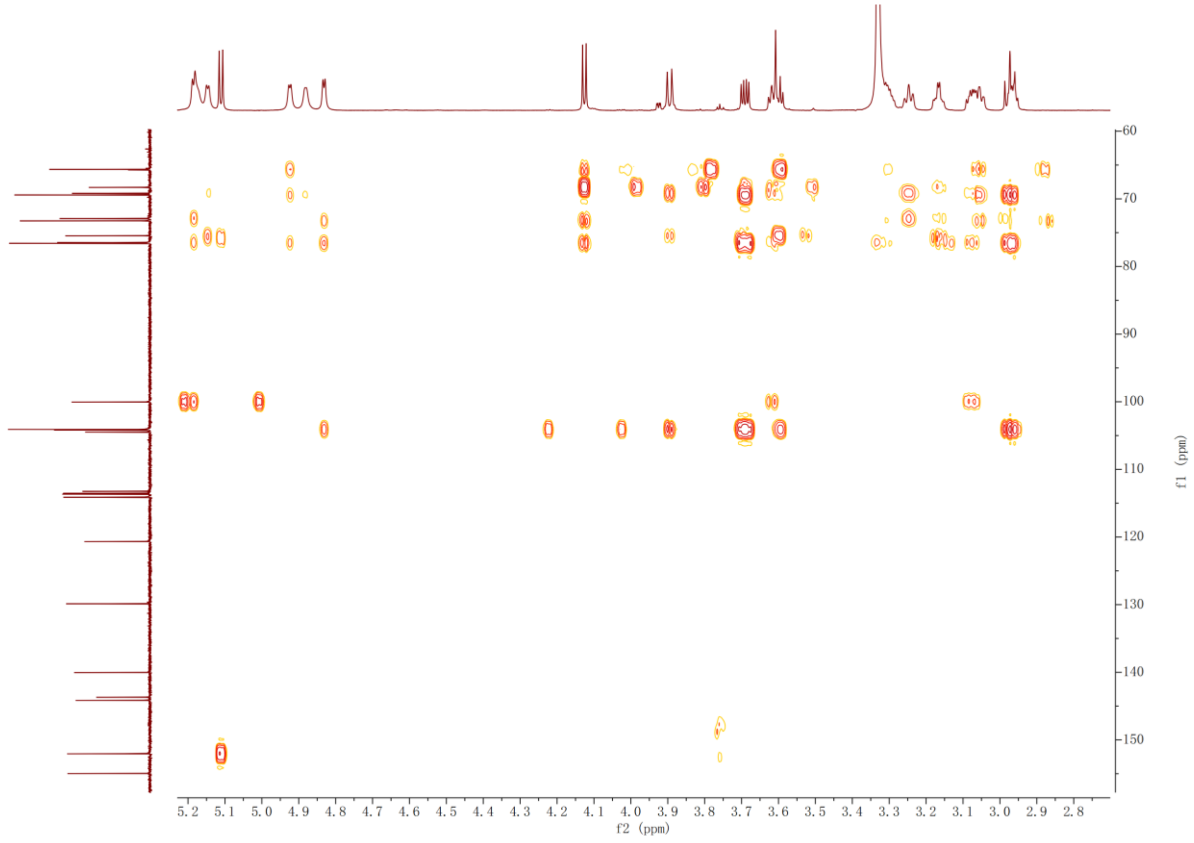

Figure S32: HMBC spectrum of compound **1** (DMSO-d_6) (expanded).

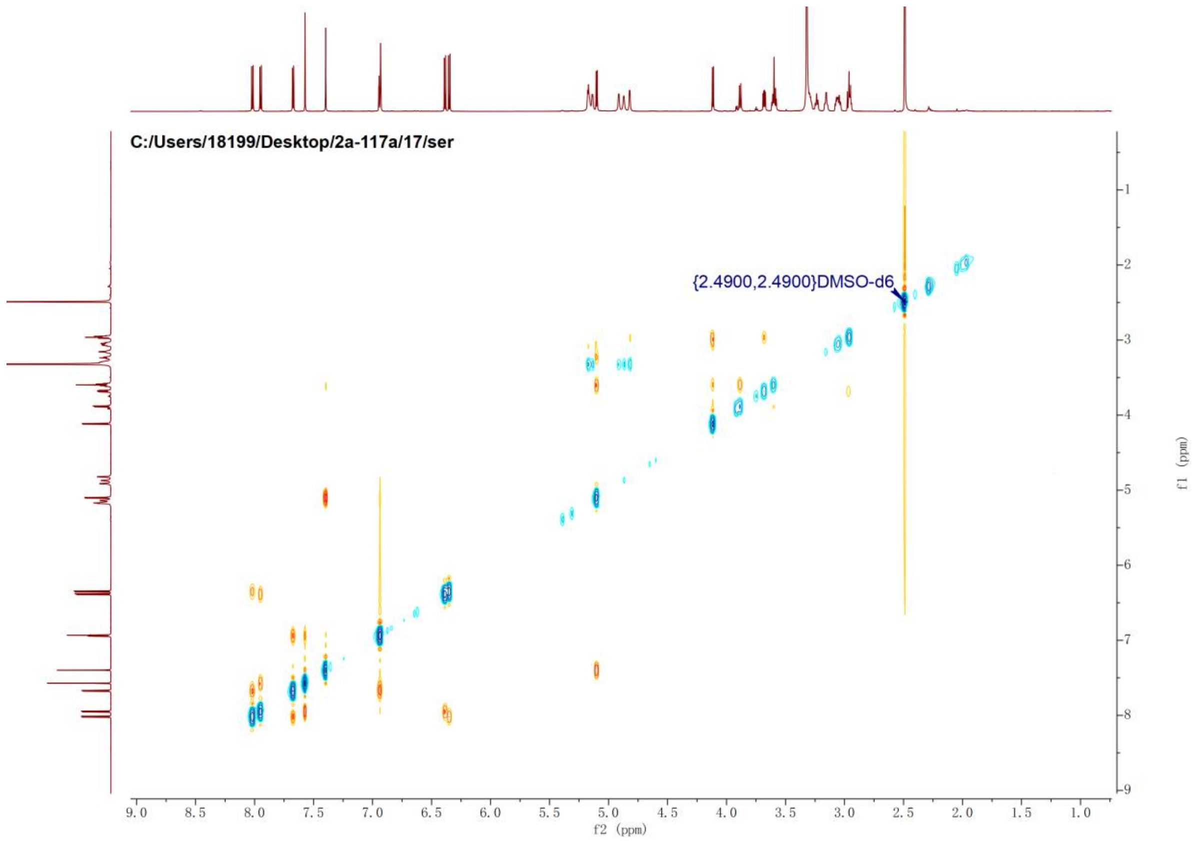

Figure S33: ROESY spectrum of compound **1** (DMSO-d_6).

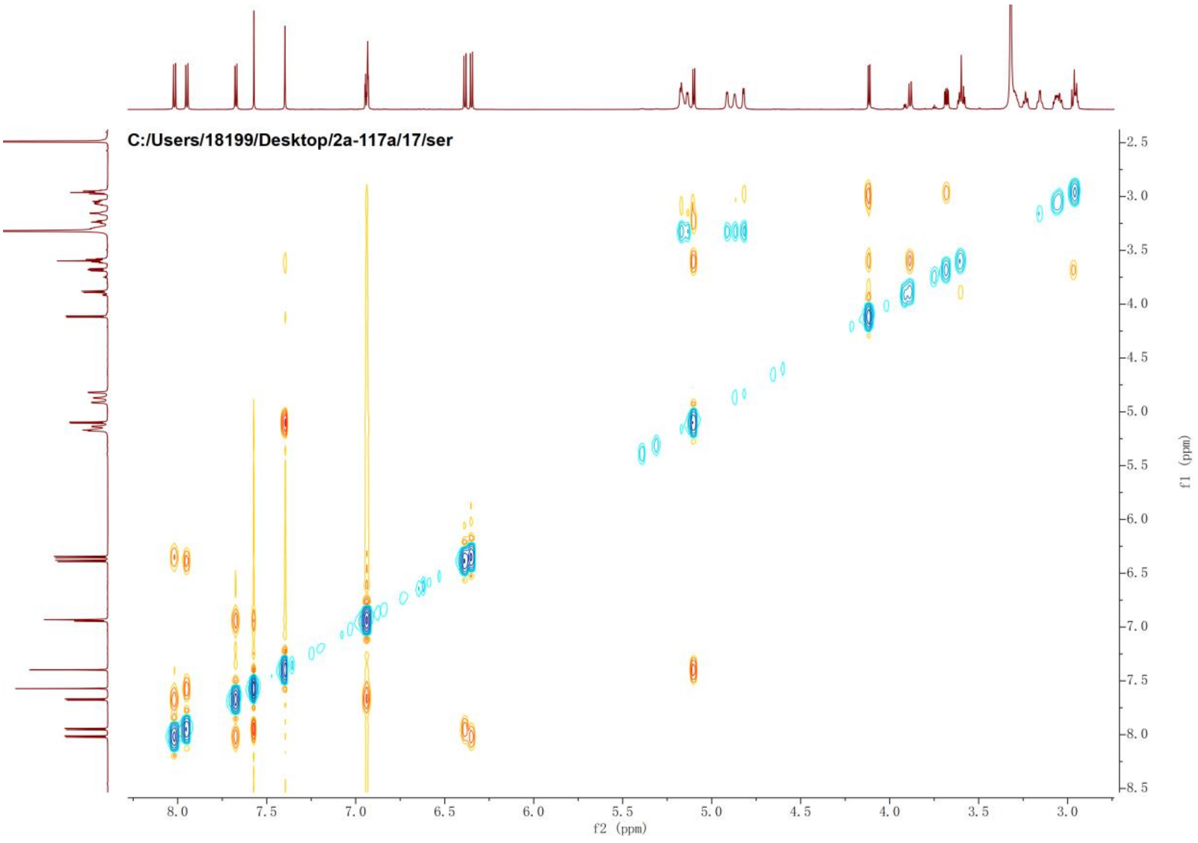

Figure S34: ROESY spectrum of compound **1** (DMSO-d_6) (expanded).
